# Common themes in architecture and interactions of prokaryotic PolB2 and Pol V mutasomes inferred from *in silico* studies

**DOI:** 10.1016/j.csbj.2025.01.010

**Published:** 2025-01-16

**Authors:** Kęstutis Timinskas, Albertas Timinskas, Česlovas Venclovas

**Affiliations:** Institute of Biotechnology, Life Sciences Center, Vilnius University, Saulėtekio av. 7, Vilnius LT-10257, Lithuania

**Keywords:** DNA polymerases, Translesion DNA synthesis, Rad51, RecA, Sequence analysis, AlphaFold structural models, Model assessment

## Abstract

Translesion DNA synthesis (TLS) is typically performed by inherently error-prone Y-family DNA polymerases. Extensively studied *Escherichia coli* Pol V mutasome, composed of UmuC, an UmuD′ dimer and RecA is an example of a multimeric Y-family TLS polymerase. Less commonly TLS is performed by DNA polymerases of other families. One of the most intriguing such cases in B-family is represented by archaeal PolB2 and its bacterial homologs. Previously thought to be catalytically inactive, PolB2 was recently shown to be absolutely required for targeted mutagenesis in *Sulfolobus islandicus*. However, the composition and structure of the PolB2 holoenzyme remain unknown. We used highly accurate AlphaFold structural models, coupled with protein sequence and genome context analysis to comprehensively characterize PolB2 and its associated proteins, PPB2, a small helical protein, and iRadA, a catalytically inactive Rad51 homolog. We showed that these three proteins can form a heteropentameric PolB2 complex featuring high confidence modeling scores. Unexpectedly, we found that PolB2 binds iRadA through a structural motif reminiscent of RadA/Rad51 oligomerization motif. In some mutasomes we identified clamp binding motifs, present in either iRadA or PolB2, but rarely in both. We also used AlphaFold to derive a three-dimensional structure of Pol V, for which the experimental structure remains unsolved thus precluding comprehensive understanding of its molecular mechanism. Our analysis showed that the structural features of Pol V explain many of the puzzling previous experimental results. Even though models of PolB2 and Pol V mutasomes are structurally different, we found striking similarities in their architectural organization and interactions.

## Graphical Abstract

**Figure:**
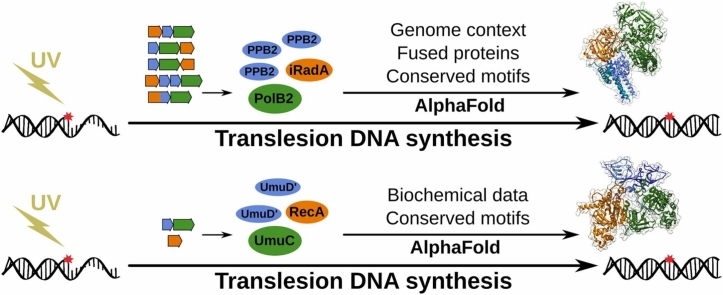

## 1 Introduction

DNA polymerases are key players in replication, repair and maintenance of genomic DNA [1]. Replicative DNA polymerases of cellular organisms belong to the three evolutionarily distinct families, C (bacteria), B (eukaryotes and some archaea) and D (archaea), and they function as part of multisubunit complexes dubbed replisomes [2]. Normally, a replisome can copy DNA with high speed and accuracy, but may stall upon encountering a DNA lesion.

Translesion DNA synthesis (TLS) most often is performed by DNA polymerases of Y-family [3]. Polymerases belonging to this family are inherently error-prone as they have a more open active site that can accommodate non-canonical or damaged base pairs and lack the proofreading 3′-5′ exonuclease activity [4], [5]. Most prokaryotic Y-family DNA polymerases correspond to a single polypeptide chain represented by *Escherichia coli* Pol IV (DinB) and its homologs in bacteria and archaea. Many bacteria, in addition to DinB homologs, have multimeric mutasome complexes exemplified by the highly mutagenic *E. coli* Pol V, composed of Y-family polymerase (UmuC), an UmuD′ dimer and RecA [6]. Pol V is very tightly regulated as it is responsible for the majority of introduced mutations upon SOS activation [4], [7], [8]. Whereas multiple structures of DinB homologs are available, the structure of Pol V mutasome, despite extensive research efforts, remains unknown, precluding comprehensive understanding of its molecular mechanism.

The majority of B-family members are replicative DNA polymerases, but organisms in all domains of life also possess B-family representatives involved in TLS synthesis. A single-subunit *E. coli* Pol II represents a SOS--inducible TLS polymerase in bacteria [9], whereas multimeric Pol ζ is a representative of B-family TLS polymerases in eukaryotes [10], [11]. Yet, arguably the most intriguing case of a B-family TLS polymerase is represented by archaeal PolB2 exemplified by Dpo2 in Sulfolobales. Initially, PolB2 was thought to represent a catalytically inactive DNA polymerase because of multiple substitutions in the active sites of its polymerase and exonuclease domains [12], [13]. However, experiments revealed that this is not the case [14]. Moreover, it was shown that PolB2 not only is an active DNA polymerase, but the only one that exhibits DNA damage-inducible expression in different Sulfolobales species [15], [16], [17], [18]. Genetic studies revealed that the B-family Dpo2 (PolB2), and not the Y-family Dpo4, is the main polymerase that mediates DNA damage tolerance and is absolutely required for targeted mutagenesis in *S. islandicus* [18]. Most recently, it was shown that Dpo2 participates in TLS primarily by extending mismatches and mispaired primer termini [19]. Although the catalytic activity of PolB2 has been characterized and its cellular role has been established, the puzzle is not yet solved. Comparative genomic analysis revealed that *polB2* and two other genes, *arCOG07300* and a *radA* homolog, belong to a putative operon suggesting that these three genes function together and that the proteins they encode may interact physically [12], [20]. Consistent with this idea, it was observed that the transcription of all three genes is strongly induced upon DNA damage [15], [16], [17]. Thus, although a purified PolB2 protein alone displays polymerase activity [19], additional data suggests that *in vivo* PolB2 functions as part of a complex with associated proteins. However, neither the composition nor the structure of the PolB2 holoenzyme have been explored.

Here, taking advantage of highly accurate protein structure prediction capabilities of AlphaFold [21], coupled with protein sequence and genome context analysis we comprehensively characterized PolB2 and its associated proteins. Furthermore, we used the expanded and thoroughly tested AlphaFold-based modeling capabilities of protein assemblies [22], [23] to show that PolB2 together with arCOG07300 (we renamed it ‘Partner of PolB2’ (PPB2)) and RadA-like proteins can form a multimeric complex. We also used protein assembly modeling to obtain complete multimeric structural models of Pol V representatives. We found that there are striking similarities in interactions and architectural organization of PolB2 and Pol V TLS complexes.

## 2 Materials and methods

### 2.1 Sequence search, clustering and gene neighborhood analysis

Initially, PolB2 homologs (B2 and G2 groups) were extracted from Supplementary Table S5 of a previous study [24]. *S. islandicus* PolB2 sequence (NCBI id: WP_014513664.1) was added for reference to this sequence set. Sequences were aligned using MAFFT in the accuracy-oriented L-INS-i mode [25]. Misaligned and truncated sequences were discarded. The resulting multiple sequence alignment (MSA) was trimmed to keep only the region containing ‘Palm’ and ‘Fingers’ domains (residues 258–421 of *S. islandicus* PolB2 sequence). The trimmed MSA was then used to search for PolB2 homologs in the NCBI non-redundant protein sequence database (ftp.ncbi.nih.gov/blast/db/) using HMMER (‘hmmsearch’ algorithm, E-value cutoff 1e-10) [26]. Taxonomy data for protein sequences were obtained from the NCBI Taxonomy database [27]. Only bacterial and archaeal sequences were retained for further analyses. Sequence clustering and grouping was done using CLANS [28]. Smaller sequence sets with lower sequence redundancy were obtained with CD-HIT [29].

Gene neighborhoods ( ± 5 genes) of the *polB2* genes were analyzed using GCsnap tool [30]. HHpred web server [31] was used to validate PPB2 and iRadA homologs within the gene neighbor groups assigned by GCsnap. Proteins encoded by the same neighborhoods were also scanned using HMMER for homologies to any of the PFAM database domains [32]. Most significant and abundant PFAM domain matches were retained for detailed manual inspection.

### 2.2 Identification and analysis of conserved protein motifs

For conservation analysis, MSAs were produced using MAFFT (L-INS-i) [25]. Jalview [33] was used for MSA analysis, editing and visualization. Sequence logos were generated using WebLogo [34]. ESPript3 [35] was used to visualize alignment conservation. To identify potential clamp binding motifs in the analyzed proteins, two separate motif MSAs (for archaea and bacteria) were generated using sequences of B1 (archaeal) and G1 (bacterial) groups obtained from Supplementary Table S5 data of a previous study [24]. First, the sequence sets were filtered to maximum 70 % sequence identity with CD-HIT, and an MSA for each set was produced with MAFFT (L-INS-i). MSA regions containing a clamp binding motif [36] were identified followed by removal of all the other regions of MSA. The resulting MSA fragment corresponding to a clamp binding motif was further inspected and any unaligned sequences were removed. The resulting motif-specific MSAs were used to search through the analyzed sequences (PolB2 and iRadA) using HMMER. Only results with the similarity score ≥ -3 and motifs identified no further than 30 residues from the sequence C-terminus were retained.

### 2.3 Phylogenetic analysis

Phylogenetic analysis was performed only for the PolB2 homologs with identified *iRadA* and *PPB2* genes in the genomic neighborhood. The PolB2 sequence set was further reduced with CD-HIT [29] to include only protein sequences that share no more than 70 % sequence identity. Fifty closest PolB3 homolog sequences (based on clustering analysis) were added to the sequence set to serve as an outgroup in the resulting phylogenetic tree. MSA was calculated using MAFFT (L-INS-i). TrimAl [37] was used to remove alignment positions that contained gaps in more than 70 % of sequences. Phylogenetic tree was constructed using IQ-tree (version 1.16.12, parameters: 1000 ultrafast bootstrap replicates (-bb 1000) and 1000 replicates for the SH-aLRT test (-alrt 1000), automatic evolutionary model selection) [38]. Analysis and visualization of the phylogenetic tree was done using iTOL [39].

### 2.4 Protein structure modeling and analysis

Monomeric structural models for the initial structural analysis were obtained from the AlphaFold Protein Structure Database (AFDB) [40]. All multimeric structural models (and PPB2 monomer models for the analysis of optimal oligomeric structure) were generated with locally installed AlphaFold-Multimer, an extension of AlphaFold2, optimized to predict multi-chain proteins [41]. Modeling was performed using default settings, default full sequence databases (‘full_dbs’) for the MSA construction and allowing use of structural templates. The best model out of five generated was selected using the default AlphaFold-Multimer ranking confidence score. Models were evaluated using AlphaFold confidence scores, VoroMQA statistical energy scores for structures and interfaces [42] and visual inspection. Structural models of DNA polymerase complexes with bound DNA were constructed by copying DNA from a known structure of close homolog after structure-based superposition of corresponding polymerase ‘Palm’ domains. The clashes between the protein chain(s) and the modeled-in DNA were minimized using the ‘minimize energy’ function in UCSF Chimera [43]. UCSF Chimera was also used for structure analysis and visualization. Additional models for protein complexes with bound DNA were generated using AlphaFold3 [44]. Searches for structural homologs were performed using the Dali server [45].

## 3 Results

We first collected B-family DNA polymerase sequences from the NCBI non-redundant sequence database and retained only archaeal and bacterial sequences. Consistent with previous studies [12], [24], sequence clustering revealed that the PolB2 group includes both archaeal and bacterial sequences and is most closely related to PolB3 and PolB1 groups of archaeal DNA polymerases (Supplementary Figure S1).

### 3.1 PolB2 is an archaeal polymerase horizontally transferred to bacteria

**Fig. 1:**
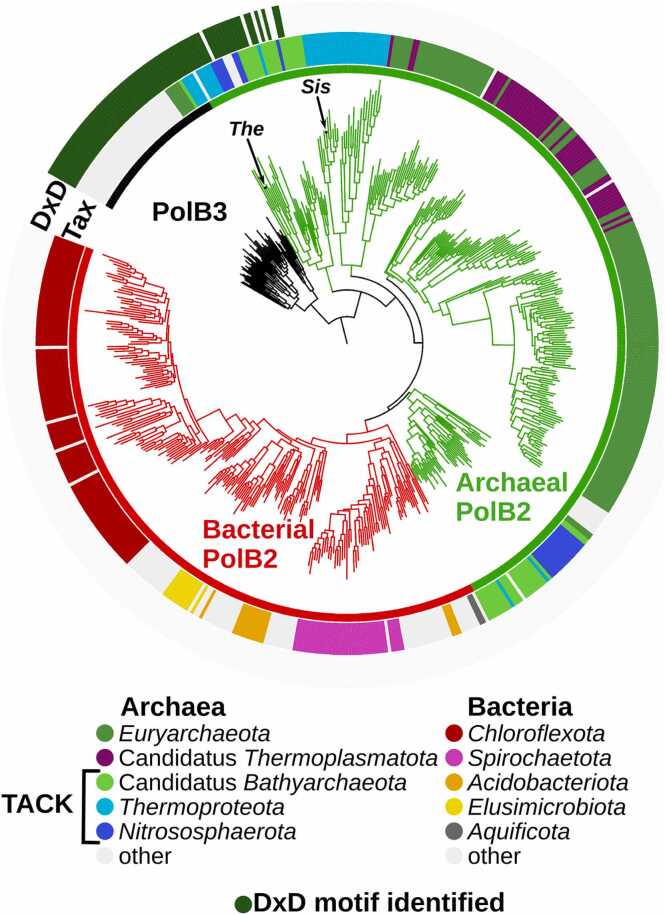
Phylogenetic tree of PolB2 and 50 closest PolB3 homologs. Tree branch colors: archaeal PolB2 (green), bacterial PolB2 (red), PolB3 outgroup (black). Middle ring (‘Tax’) shows the taxonomy (color legend provided below the tree). The outer ring (‘DxD’) shows the presence (green) or absence (grey) of the canonical DxD motif of the polymerase active site. ‘*Sis’* – *S. islandicus* PolB2 (no DxD motif), ‘*The’* - *Thermoprotei* archaeaon PolB2 (with the DxD motif).

To explore the PolB2 group in detail, we identified putative PolB2 operons and performed phylogenetic analysis of PolB2 sequences belonging to these operons. We constructed the PolB2 phylogenetic tree using PolB3 as an outgroup (Fig. 1). In agreement with a previous study [24], the tree suggests that PolB2 evolved in archaea and was subsequently introduced into bacteria. A clear separation between archaeal and bacterial sequences suggests that PolB2 was introduced into bacteria through a single horizontal transfer event.

The majority of PolB2 sequences bear substitutions of the first conserved aspartate within the canonical polymerase active site motif (DxD), the primary cause of previous erroneous predictions of the catalytic inactivation [12], [13]. However, to our surprise, we found that some PolB2 sequences do have the intact DxD motif (Fig. 1). Interestingly, all these sequences appear in the archaeal clade, closest to PolB3, and originate from the TACK (Thaumarchaeota, Aigarchaeota, Crenarchaeota and Korarchaeota) superphylum [46]. Nonetheless, not all TACK archaea have PolB2 sequences with the canonical DxD motif. Moreover, PolB2 sequences from the TACK group appear in different clades in the PolB2 tree suggesting frequent horizontal transfers of PolB2 within archaeal species, which contrasts with the proposed single transfer event between archaea and bacteria.

**Fig. 2:**
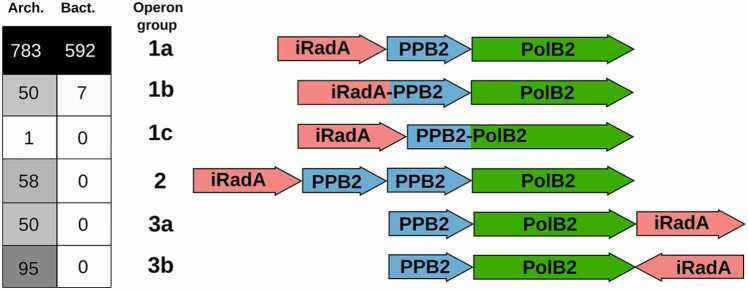
PolB2 operon groups and their distribution in the analyzed genomes. Only groups with the genes coding for iRadA, PPB2 and PolB2 in the vicinity of each other are shown.

Our analysis of putative PolB2 operons substantiates previously discovered tight association of PolB2 with iRadA (a RadA homolog with inactivated ATPase active site) and PPB2 (previously arCOG07300) [12]. The corresponding three genes in most cases are arranged in the same orientation next to each other and only rarely interspersed with other genes. This observation supports the notion that the three genes belong to a single operon and that proteins encoded by these genes may form a multimeric complex. We observed several major variants of the gene arrangement order (Fig. 2; Supplementary data file 1). In some cases, we observed fusions between two of the components further supporting the idea that proteins encoded by the PolB2 operons interact physically. Notably, whereas archaea have all the variants of putative operons, bacteria possess only operons in which the genes are arranged in the order *iRadA-PPB2-PolB2* including those that are interspersed with other genes or featuring *iRadA-PPB2* gene fusions (Supplementary Figure S2). The only order of the three genes observed in bacterial operons further supports a scenario, in which an entire archaeal PolB2 operon was transferred to bacteria and has since undergone some diversification (fusion of iRadA with PPB2 or insertion of additional genes within the operon).

As it has been previously reported that the expression of PolB2 operons is induced upon DNA damage [15], [16], [17], [18], [19], we explored whether the immediate neighborhood contains putative transcription regulators or genes associated with the DNA damage processing. Consistent with previous observation [12], we found that the genomic neighborhoods of *polB2* are enriched with *orc1/cdc6* homologs and *lexA* in archaea and bacteria, respectively (Supplementary Table S1, Supplementary data file 1). This observation aligns well with the DNA damage inducible nature of PolB2 operons, as LexA is a key regulator of the SOS system in bacteria [7], whereas Orc1/Cdc6 homologs were recently found to play a key role in DNA-damage response in archaea [17]. In addition, we identified two other genes, apparently associated with DNA damage response, enriched to some degree in the neighborhood of *polB2* in both archaea and bacteria. One of these genes codes for SRAP [47], a protein known to be involved in the protection of DNA abasic sites [48]. The second gene codes for a DUF72 family protein, for which the function has not yet been assigned. However, structures of several DUF72 representatives have been solved (PDB ids: 1VPQ, 1VPY, 1ZTV). DUF72 proteins have the TIM-barrel structure, which is closely related to the UV-damage endonuclease, a DNA-repair enzyme that can recognize and incise different types of damaged DNA [49].

### 3.2 Structural models reveal differences of PolB2 and iRadA from their homologs and suggest the trimeric state for PPB2

**Fig. 3:**
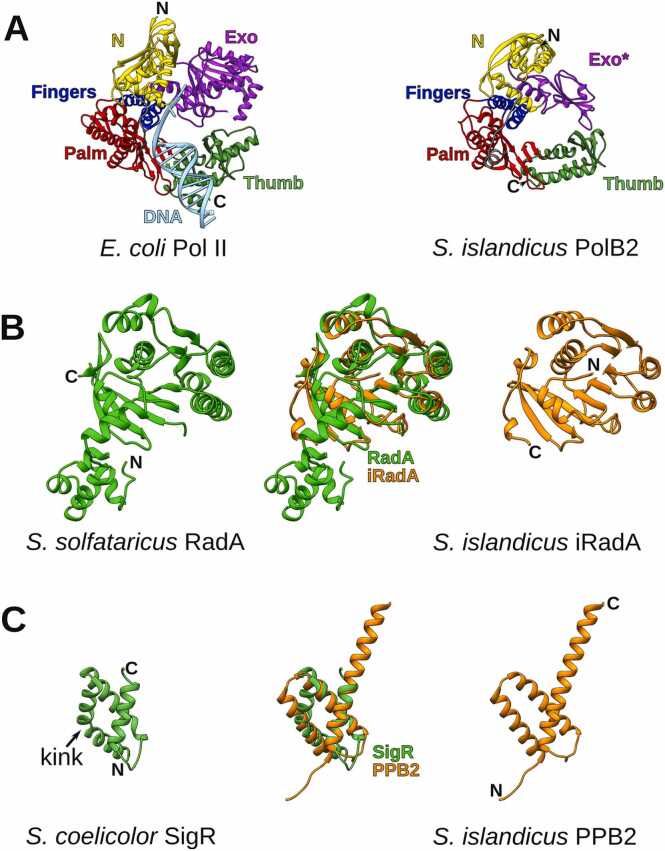
Structural relationships of PolB2, iRadA and PPB2 models. (A) Comparison of *E. coli* Pol II (PDB id: 3k59) and *S. islandicus* PolB2 (AFDB id: F0NED6) structures (493 residue pairs can be superimposed with 4.8 Å RMSD). Corresponding domains are colored using the same colors. ‘Exo* ’ indicates that the exonuclease domain of PolB2 lacks the active site. (B) Comparison of *S. solfataricus* RadA (PDB id: 2zub) and *S. islandicus* iRadA (AFDB id: F0NED7). Superimposed structures (161 residue pairs) produce 2.4 Å RMSD. (C) Comparison of *S. coelicolor* N-terminal domain of σ-factor SigR (PDB id: 1h3l) and *S. islandicus* PPB2 (AFDB id: F0NED5) monomer (72 residue pairs, 3.2 Å RMSD).

Initially, we explored predicted structures of PolB2, iRadA and PPB2 proteins. We retrieved structural models for individual *S. islandicus* proteins from the EBI AlphaFold database [40]. The structure of PolB2 is fairly similar to that of a canonical DNA polymerase of B-family, except that the proofreading exonuclease domain lacks the active site and shows various degrees of structural decay (Fig. 3A, Supplementary Figure S3). Interestingly, the level of decay of proofreading exonuclease seems to correlate with the evolutionary distance between PolB2 and PolB3. For example, *Thermoprotei* archaeon PolB2, positioned close to the root of phylogenetic tree and featuring the canonical polymerase active site motif (DxD), has a more structurally complete proofreading domain than that of the *S. islandicus* PolB2, which is more distantly related to PolB3 and has Ile instead of the first Asp (401-IID-403) (Fig. 1, Supplementary Figure S3).

Another component, iRadA, shows close similarity to the archaeal Rad51 homolog (RadA), eukaryotic Rad51 and to a lesser extent to bacterial RecA (Fig. 3B, Supplementary Figure S4). The major difference is that iRadA lacks the N-terminal helical domain, involved in oligomerization of RadA (Rad51) and that the ATPase active site residues are missing (Supplementary Figure S5).

PPB2 is a small α-helical protein consisting of three helices. Using Dali [45], we found that the closest structural matches in PDB correspond to the N-terminal domain of various σ-factors (top Dali Z-score = 7.7). The major difference is in that the first α-helix in σ-factors has a typical kink [50], whereas the corresponding PPB2 helix is straight (Fig. 3C). As this relationship could be detected only at the structural level, it is unclear whether PPB2 and the N-terminal domain of σ-factors are evolutionarily related. Surprisingly, the evaluation of a PPB2 monomeric structure revealed that despite very high AlphaFold confidence score (pLDDT > 90), the structure has very low global VoroMQA score (<0.3) indicating that it is energetically unfavorable. We also noticed that PPB2 does not form a typical globular structure with a hydrophobic core. Instead, some of the hydrophobic residues are exposed on the surface. Collectively, these observations suggested that PPB2 may exist as an oligomer. Therefore, we explored possible oligomeric states of *S. islandicus* PPB2 by modeling and assessing a monomer, a dimer, a trimer and a tetramer. Indeed, we found that the trimer, and not the monomer or another oligomer, represents the most favorable energy state and has the best AlphaFold-Multimer scores (Supplementary Figure S6). To make sure that this result is not specific only to *S. islandicus* PPB2, we additionally selected 39 diverse archaeal and bacterial PPB2 proteins and performed the same computational experiment. The results of this experiment confirmed that the trimer is the optimal oligomeric state for PPB2 (Table 1; Supplementary data file 2). The PPB2 trimer represents a coiled coil pillar topped by a wider helical capital formed by the N-terminal regions (Supplementary Figure S6).

**Table 1:** Average quality values of PPB2 structural models in different oligomeric states.

**PPB2 oligomer**^a^	**AF pLDDT**^b^	**AF pTM**^b^	**AF ranking score**^b^	**VoroMQA score**^b^	**VoroMQA i-score**^b^
monomer	86 ± 2	0.72 ± 0.03	-	0.28 ± 0.01	-
dimer	78 ± 3	0.71 ± 0.04	0.69 ± 0.05	0.44 ± 0.01	0.57 ± 0.01
trimer	**89 ± 2**	**0.88 ± 0.02**	**0.87 ± 0.02**	**0.53 ± 0.01**	**0.65 ± 0.01**
tetramer	65 ± 3	0.57 ± 0.04	0.54 ± 0.05	0.49 ± 0.01	0.59 ± 0.02

a Values for each oligomeric state were derived from 40 models.

b The highest values are shown in bold. Error values were calculated as 95 % confidence score, based on t-distribution of sample standard deviation.

### 3.3 PolB2 complex with iRadA and PPB2 reveals novel interaction modes

**Fig. 4:**
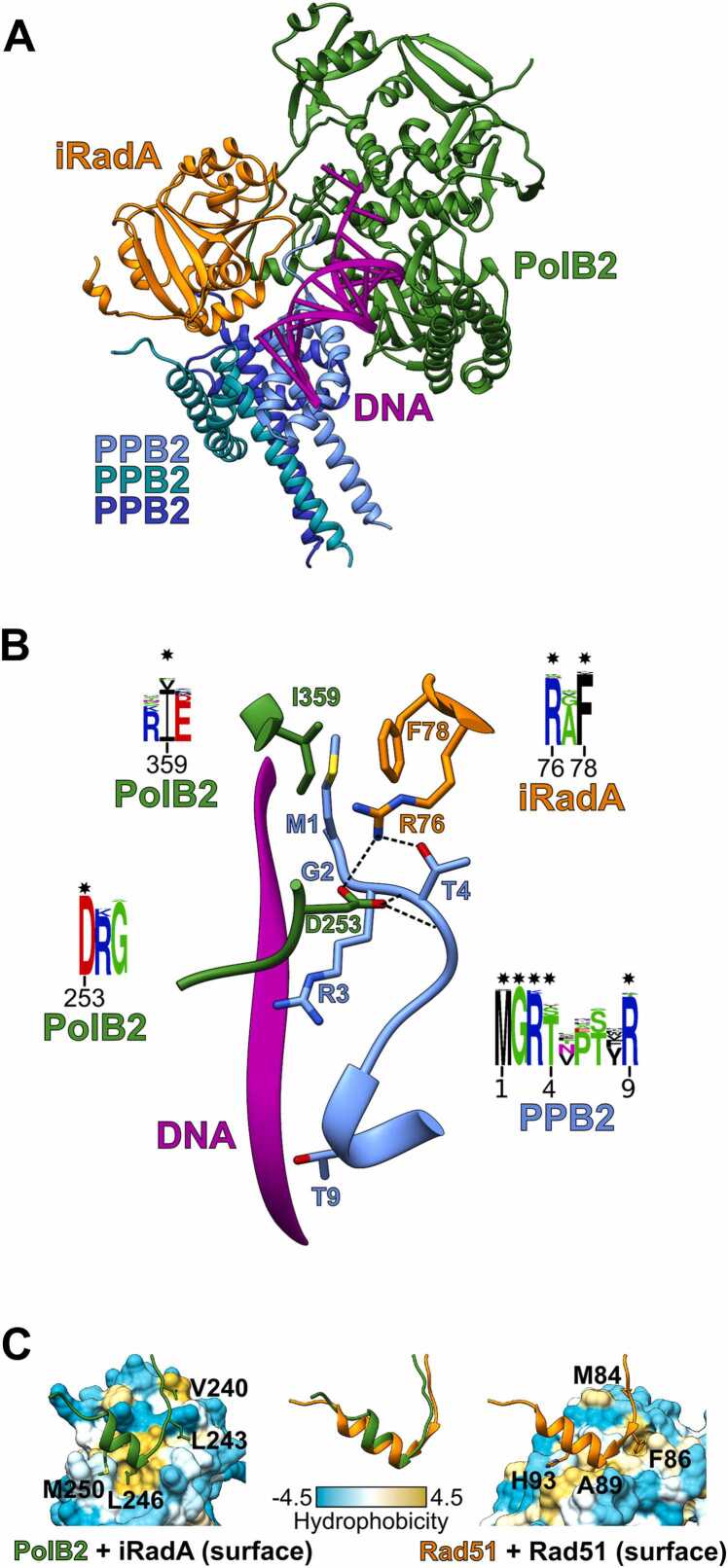
Structure and conserved interactions of the PolB2 complex. (A) Full model of *S. islandicus* heteropentameric PolB2 complex. Modeled-in DNA was copied from *T. kodakarensis* PolB1 structure (PDB id: 4k8z) after structural superposition of corresponding ‘Palm’ domains. (B) Conserved interactions between the PPB2 N-terminal tail and other components of the PolB2 complex. Conserved interactions are indicated with dashed lines, whereas interacting conserved residues are indicated with stars in sequence logos. (C) Comparison of *S. islandicus* PolB2-iRadA interaction (model) and human Rad51-Rad51 interaction (PDB id: 8pbc). PolB2 motif mediating the interaction with iRadA is shown as a green ribbon and the corresponding Rad51 motif is in orange. iRadA and Rad51 are shown as molecular surfaces, colored by hydrophobicity.

In the following step, we proceeded to computationally test whether PolB2, iRadA and PPB2 may form a multimeric complex. Having established that the trimer is an optimal oligomeric state for PPB2 proteins, we used three copies of PPB2 and single copies of both PolB2 and iRadA to model putative complexes. To increase the reliability of results, we generated corresponding models for 40 putative complexes spanning diverse archaeal and bacterial species. All generated pentameric structural models were of high confidence (AlphaFold ranking scores ranging from 0.8 to 0.91 with the mean value of 0.86) and showed favorable energy scores (Supplementary data file 2). We also tested whether models produced by the newly released AlphaFold3 were consistent with those produced by AlphaFold-Multimer. Indeed, both methods produced closely similar models further supporting the reliability of these computationally derived structures. Fig. 4A shows a structural model of *S. islandicus* multimeric PolB2 heteropentamer in complex with DNA obtained using AlphaFold-Multimer, and its comparison with an AlphaFold3-generated model is provided in Supplementary Figure S7.

#### 3.3.1 PPB2 binding is mediated via its N-terminal arm

PPB2 forms a symmetric trimer, but it interacts with PolB2 asymmetrically. The N-terminal arm of one monomer is inserted into the cleft formed by PolB2 and iRadA and is also close to the modeled-in DNA (Fig. 4B). In the model this N-terminal arm, particularly the four highly conserved N-terminal residues (the ‘MGRT’ motif), participates in multiple conserved interactions with both PolB2 and iRadA. Most likely it also contributes to DNA binding through the two conserved Arg residues (positions 3 and 9), one of which is replaced with Thr9 in *S. islandicus* (Fig. 4B). The observed interactions imply the importance of the N-terminal arm of PPB2 for the function and/or stability of the entire PolB2 complex. Consistent with this idea, we found that several N-terminal residues of PPB2 are highly conserved, both in sequences and structures (Fig. 4B, Supplementary Figure S8). Interestingly, in some operons PPB2 is fused to iRadA (in one case to PolB2), in some others there is a duplication of the PPB2 gene (Fig. 2) raising a question of what happens in such cases. Upon closer inspection, we found that mRNAs of fused iRadA-PPB2 show enrichment of Shine-Dalgarno motifs upstream of the PPB2-coding region suggesting that both the iRadA-PPB2 fusion and individual PPB2 can be produced from the same mRNA (Supplementary Figure S9). Notably, the PPB2 sequences derived from the fusion also feature the conserved N-terminal region. As to the PPB2 duplications, we found that in all such operons only one PPB2 copy possesses the N-terminal ‘MGRT’ motif, while the other lacks it (Supplementary Figure S10). We performed multiple modeling experiments and established that these two copies can form both homotrimeric and mixed heterotrimeric models with similarly high confidence. However, the most reliable multimeric PolB2 complexes were obtained in cases, when the PPB2 trimer consisted of just one copy of PPB2 with the conserved N-terminal arm (see Supplementary Figure S11, Supplementary Table S2 and Supplementary data file 2 for details). Thus, it appears that this highly conserved arm is a key element in the formation of PolB2 complex.

#### 3.3.2 PolB2-iRadA interaction is similar to the Rad51/RadA interaction within the filament

In our previous study [24] we observed that PolB2 sequences have a conserved D(K/R) motif (253-DK-254 in *S. islandicus* PolB2) in a flexible loop and hypothesized that the conserved Asp might replace the ‘missing’ first Asp in the DxD motif (401-IID-403 in *S. islandicus* PolB2). However, the AlphaFold model revealed that the role of this motif is different as it interacts with the conserved N-terminal arm of PPB2 (Fig. 4B). On the other hand, the structural role of the region preceding the conserved ‘DK’ motif turned out to be most unexpected. We found that this region is interacting with iRadA in a manner, which is strikingly similar to the interaction between eukaryotic Rad51 or archaeal RadA subunits in a Rad51/RadA filament [51], [52]. In the modeled complex the PolB2 loop forms a β-α motif, analogous to the Rad51/RadA oligomerization motif, which is located between the N-terminal DNA-binding domain and the central ATPase domain (Fig. 4C, Supplementary Figure S3). We also tested whether PolB2 may form a complex with RadA, an active recombinase, but PolB2 complex with RadA produced very poor AlphaFold-Multimer ranking score (0.28) and VoroMQA interface score (0.19) (see Supplementary data file 2 for details). Collectively, these results indicate that PolB2 binding is specific to iRadA.

#### 3.3.3 Multiple PolB2 complexes have clamp binding motifs

**Fig. 5:**
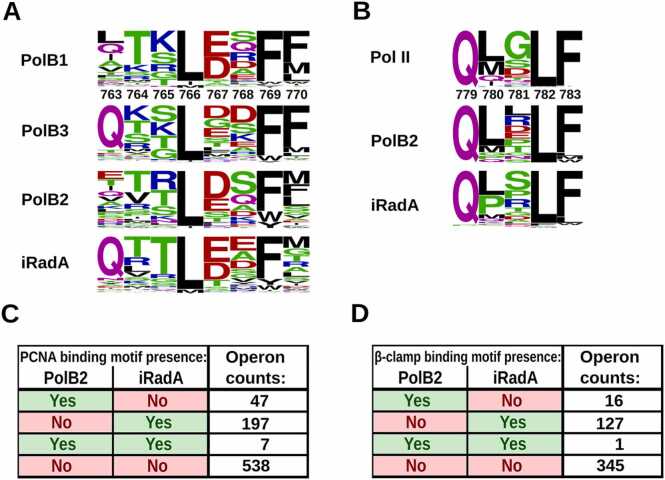
Clamp binding motifs and their presence in PolB2 and iRadA. Sequence logos for (A) PCNA binding motifs within archaeal B-family polymerases and iRadA proteins and (B) β-clamp binding motifs within bacterial B-family polymerases and iRadA proteins. Positional numbering of archaeal motifs is based on *Pyrococcus furiosus* PolB (PDB id: 3a2f; PolB complex with PCNA). Positional numbering of bacterial motifs is based on *Escherichia coli* Pol II (PDB id: 3k57). Distribution of motifs within (C) archaeal and (D) bacterial operons with at least PolB2 and iRadA coding genes identified. Sequence logos and motif distribution data were derived from protein sets reduced to no greater than 90 % sequence identity.

Many DNA polymerases, including members of the B-family, function by binding to the DNA sliding clamp to increase the affinity to DNA and processivity of the polymerase [53], [54]. We asked whether PolB2 multimeric complexes (mutasomes) may also utilize DNA sliding clamps. To this end we constructed a sequence profile for the PCNA binding motif from the alignment of PolB1 polymerases that are known to work with PCNA [55]. We then used HMMER to query PolB2 sequences with this profile for the presence of PCNA binding motif in their C-terminal regions. Indeed, we found that a small fraction of PolB2 polymerases do have PCNA binding motifs. During inspection of structural models for putative PolB2 mutasomes we noticed that in some of them the C-terminal region of iRadA is also positioned in such a way that it could bind PCNA. We performed the same search against archaeal iRadA sequences and, to our surprise, detected PCNA binding motifs in multiple iRadA sequences (Fig. 5A, C; Supplementary data file 1). Even more surprising was the observation that the number of PCNA binding motifs was significantly larger in iRadA sequences compared to PolB2 and the motifs in iRadA were somewhat closer to the canonical ‘QxxLxxFF’ motif [36]. In principle, both PolB2 and iRadA within the same mutasome could bind PCNA, but we found that PolB2 and iRadA from the same species rarely have PCNA binding motif in both (Fig. 5C). It does not, however, exclude the possibility that we may have missed many of the ‘weak’ (less similar to the canonical one) PCNA binding motifs.

The bacterial DNA sliding clamp (β-clamp) is only distantly related to PCNA. Moreover, the canonical motif (QLxLF) for binding the β-clamp is different [36]. Therefore, we were curious as to the fate of PolB2 mutasome complexes after their horizontal transfer from archaea to bacteria. To explore putative clamp binding motifs, we performed analogous computational experiment. We derived β-clamp binding motif from the aligned *E. coli* Pol II homologs and searched with this profile against bacterial PolB2 and iRadA sequences. As with archaeal sequences we found a similar pattern, that is either PolB2 or iRadA has a β-clamp binding motif, but these motifs are more frequent in iRadA and almost never found in both PolB2 and iRadA simultaneously (Fig. 5B, D; Supplementary data file 1). This observation implies that once transferred to bacteria, clamp binding motifs evolved to match the cognate bacterial clamps.

**Fig. 6:**
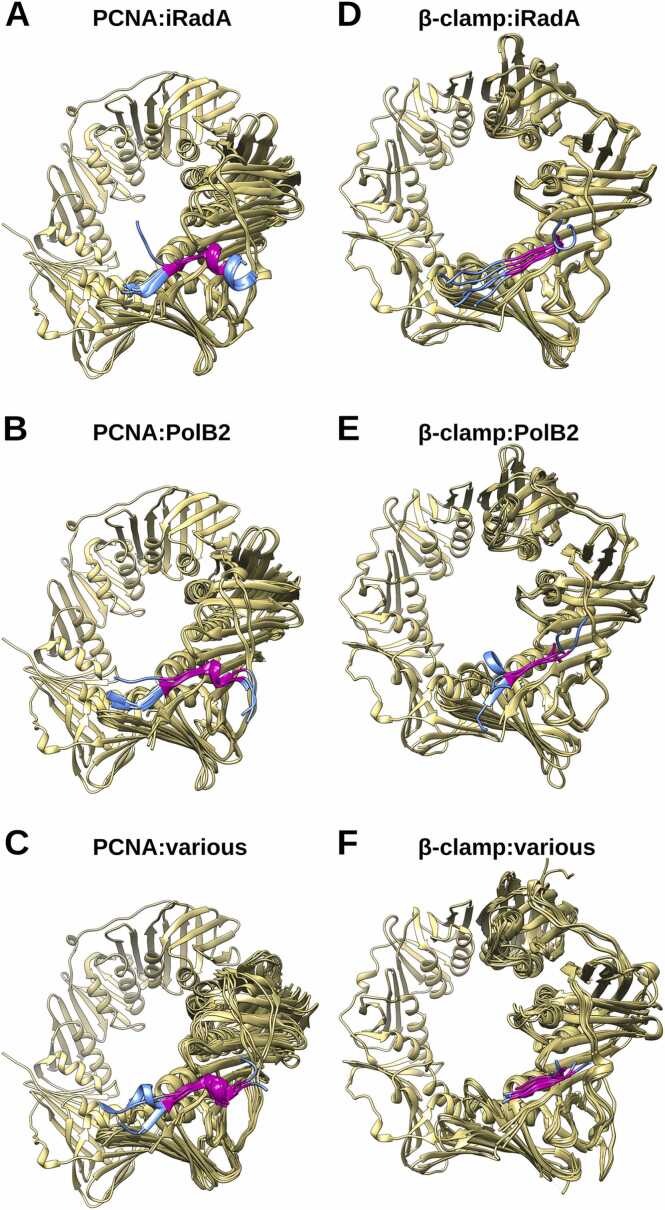
Comparison of clamp binding motifs bound to a corresponding DNA sliding clamp: (A-C) archaeal PCNA, (D-F) bacterial β-clamp. Clamp structures are colored dark khaki, clamp binding motifs are shown in magenta with residues flanking the motif shown in blue (the rest of the structure is not shown). Detailed lists of representatives are provided in the annotation of Supplementary Fig. S12.

To test whether these clamp binding motifs indeed may mediate interaction between the PolB2 mutasomes and DNA sliding clamps, we generated models for a number of such complexes. The models had high confidence scores (Supplementary data file 2) and the clamp binding motifs of PolB2 or iRadA were bound to the corresponding clamps similarly as in experimentally determined structures (Fig. 6). Additional models of PolB2 mutasomes bound to DNA and clamps derived using AlphaFold3 have further substantiated the results obtained with AlphaFold-Multimer (Supplementary Figure S12).

Thus, to summarize, the sequence and structure analyses suggest that PolB2 mutasomes consist of PolB2, iRadA, and a PPB2 trimer. The highly conserved N-terminus of PPB2 is important for the interaction with PolB2, iRadA and likely, the DNA. Another component, iRadA, interacts with PolB2 in a manner analogous to the way recombinases Rad51/RadA interact with each other within the Rad51/RadA filament. The site of iRadA involved in the interaction with PolB2 involves one of the most conserved sequence positions, strongly supporting the predicted interaction mode. Finally, both sequence and structure-based analyses indicate that at least a fraction of PolB2 mutasomes both in archaea and in bacteria function together with the cognate DNA sliding clamps.

### 3.4 Structural models of *E. coli* Pol V mutasome and its homologs rationalize known experimental data

All these computational findings hinted at possible parallels between a putative PolB2 mutasome and a bacterial Pol V mutasome, consisting of a Y-family DNA polymerase (UmuC), a dimer of an accessory subunit (UmuD′), and a Rad51 homolog in bacteria (RecA). We have earlier discovered that UmuC and many other Y-family polymerases in their C-terminal region have a sequence motif, homologous to the N-terminal RecA oligomerization motif [56]. We named this motif RecA-NT and showed that the experimental data on *E. coli* UmuC-RecA interaction strongly support our proposed mode of interaction. However, at the time our structural model covered only the UmuC RecA-NT and RecA interaction.

Here, we extended our computational study on Pol V to be able to contrast and compare structural organization of PolB2 and Pol V mutasomes. In addition to *E. coli* Pol V [6], we selected experimentally characterized highly mutagenic Pol V homologs, *rumAB* and *mucAB*, encoded by mobile genetic elements [57], [58], [59] and several other chromosomally encoded Pol V homologs. Modeling results showed that most models of Pol V homologs have high confidence scores indicating their reliability (Supplementary data file 2). Moreover, structural comparison of models not only revealed similarity between homologous subunits, but also consistently reproduced similar interaction interfaces. Therefore, for detailed analysis we selected *E. coli* Pol V, which is the most extensively studied DNA polymerase among Pol V homologs.

**Fig. 7:**
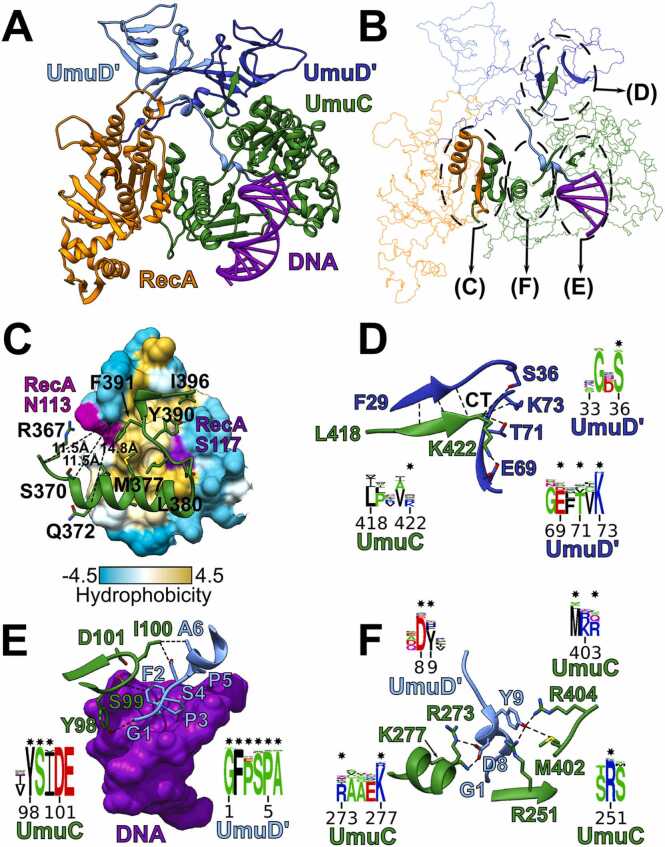
Full heterotetrameric structural model of *E. coli* Pol V and important interactions of its components. (A) A model of Pol V complex with modeled-in DNA (purple). DNA was copied into Pol V from the structure of *E. coli* Pol IV (PDB id: 5yuu) after structural superposition of corresponding ‘Palm’ domains. RecA N-terminal motif (1−37) and unstructured C-terminal residues (335−353) are not shown. (B) Wireframe representation of Pol V structure with important interaction sites (presented in detail in (C-F)) shown as ribbons. (C) Interaction of the UmuC RecA-NT motif with RecA, depicted as a surface colored by hydrophobicity. RecA N113 and the three UmuC cross-linking partners (R367, S370 and Q372) are indicated and corresponding distances between Cα atoms are displayed as dashed lines. RecA S117 that is also known to participate in the RecA-UmuC interface is colored purple. (D) UmuC C-terminal (CT) interactions with one of the UmuD’ monomers. Four residues at the very C-terminus of UmuC form a β-sheet with UmuD’ (putative H-bonds shown in dashed lines). The C-terminal carboxyl group of K422 could potentially form hydrogen bonds with multiple UmuD’ side chains (S36, T71, K73), whereas the side chain could form a salt bridge with E69 of UmuD’. (E, F) Conserved interactions of the N-terminal arm of another UmuD’ with the ‘Palm’, ‘Little finger’ domains, the C-terminal tail of UmuC and DNA. For each of the indicated structural motifs, sequence conservation is displayed as corresponding sequence logos. Putative interactions are displayed as dashed lines within the structures and the corresponding sequence positions are marked with stars above the logos.

Our computational model of *E. coli* Pol V represents a complete heterotetrameric structure that includes UmuC, RecA and 2 copies of UmuD′. We also modeled-in the DNA from the X-ray structure of *E. coli* polymerase IV after superposition of corresponding ‘Palm’ domains (Fig. 7A). Additionally, we applied modeling with AlphaFold3 to make sure that results are consistent. AlphaFold3 produced a highly similar model, thus further boosting the confidence in the computationally derived structure of Pol V (Supplementary Figure S13). Analysis of the Pol V model corroborated our previously proposed UmuC-RecA interaction mode (Fig. 7C). In the model UmuC interacts with RecA via the RecA-NT motif and, as discussed extensively in our previous study [56], this interaction is directly supported by the cross-linking data [60]. The very UmuC C-terminal region (L418-K422) binds to one UmuD′ monomer by providing an additional β-strand at the edge of antiparallel six-stranded β-sheet formed by the UmuD′ dimer (Fig. 7D). This interaction suggests that the C-terminal fragment of UmuC plays a critical role in UmuD′ binding. Indeed, it was demonstrated that the removal of the last 26 UmuC residues eliminates the ability of UmuC to interact with UmuD′ [61], [62] and to perform SOS mutagenesis [62]. Moreover, it was shown that the deletion of just one C-terminal residue of UmuC significantly reduced levels of Pol V-dependent spontaneous mutagenesis, while mutants lacking two or three C-terminal residues of UmuC were rendered essentially non-mutable [8]. In addition, removal of even a single residue from the UmuC C-terminus in the Pol V context made UmuC susceptible to fast degradation by Lon protease, whereas wt UmuC remained intact [8]. It can be seen in the model that the removal of one or more residues from the C-terminus of UmuC would compromise the UmuC interaction with UmuD′. Particularly, the very C-terminus of UmuC appears to be positioned to form multiple potential hydrogen bond/salt bridge interactions (Fig. 7D).

The two N-terminal arms of the UmuD′ dimer are in very different structural contexts. One arm is exposed to the solvent, whereas the second one binds UmuC in such a way that the highly conserved N-terminal end of UmuD′ is positioned close to the active site of UmuC and potentially might contact bound DNA (Fig. 7E, F; Supplementary Figure S14). Furthermore, it forms multiple conserved contacts with both ‘Palm’ (near the active site) and ‘Little finger’ domains as well as the C-terminus of UmuC. This arrangement implies that the properties of the UmuD′ N-terminal arm are important for the Pol V function. Consistent with this implication, an armless UmuD′ was shown to be proficient in forming a dimeric structure, yet completely defective in its ability to support SOS-dependent spontaneous or methyl methanesulfonic acid (MMS) induced mutagenesis [63]. Furthermore, it was shown that in the absence of RecJ processing of DNA lesions, when both the recovery and cell survival become dependent on translesion synthesis by Pol V [64], the removal or substitution of a single N-terminal residue of the UmuD′ arm has a pronounced effect on the UV-induced mutagenesis by both wt and A9V UmuC mutant [65]. In the model Ala9 is not directly involved in interaction with UmuD’, but is in the vicinity of the UmuC active site and the ‘Fingers’ domain (Supplementary Figure S15). On the other hand, an exposed position of the second UmuD′ arm is consistent with cross-linking experiments, showing that within the Pol V complex RecA at the F21 position can be cross-linked to UmuD′ but not to UmuC [66]. Indeed, in our Pol V mutasome model, both RecA and UmuD′ N-terminal regions are exposed to the solvent and may approach each other unhindered, whereas the closest approach to UmuC is partially obstructed by the core domain of RecA and the distance between them is larger (Supplementary Figure S16).

Pol V mutasome is known to function with β-clamp [67]. Therefore, we asked whether the interaction with β-clamp could be reproduced in a computational model. For this, we used AlphaFold3 to generate a model of Pol V complexed with both DNA and the β-clamp (Supplementary Figure S17). In the model, UmuC closely reproduced the experimentally determined interaction mode with the β-clamp [68].

Taken together, all these experimental observations regarding Pol V function and interactions provide overwhelming support to the structural model of Pol V presented here.

## 4 Discussion

Putative PolB2 mutasome, characterized here computationally, represents the first multimeric prokaryotic B-family TLS polymerase. Our results imply that *in vivo* PolB2 forms a heteropentameric complex, composed of the catalytic subunit, an inactive RadA homolog (iRadA) and a trimer of a small helical protein (PPB2). The formation of multimeric PolB2 complex is strongly supported by several lines of data: (1) tight coupling of the three genes in corresponding genomes, (2) coordinated induction of their transcription, (3) observed iRadA-PPB2 and PPB2-PolB2 fusions, and most importantly (4) by multiple high-confidence structural models representing diverse archaeal and bacterial species. At first glance the *in vitro* experimental results showing that the PolB2 catalytic subunit alone is active as a TLS polymerase [19] may seem at odds with our computational results. However, the data coming from studies of yeast Pol ζ, suggest that there might be no contradiction. Pol ζ is a heteropentameric complex, composed of the catalytic subunit, Rev3, a dimer of accessory subunit Rev7 and two other subunits, Pol31, and Pol32, shared with replicative polymerase Pol δ [11]. A recent study has shown that yeast Pol ζ, which lacks the Rev3 C-terminal domain serving as the platform for interaction with Pol31 and Pol32, retains most Pol ζ functions [69]. In the case of PolB2, the situation might be analogous. In other words, the properties of the isolated PolB2 protein *in vitro* may well reflect the *in vivo* properties of the multimeric PolB2 mutasome. The similarities between PolB2 and Pol ζ extend even further. Both polymerases are induced in response to DNA damage, both have inactivated 3′–5′ proofreading exonuclease domain and both function primarily as “extenders” of distorted primer termini past the lesion [11], [19].

PolB2 has much less in common with the group of bacterial TLS DNA polymerases represented by *E. coli* Pol II, which corresponds to a single subunit and has an active 3′–5′ exonuclease domain. The only seemingly common feature is that Pol II, similarly to PolB2 and Pol ζ, is primarily an “extender” polymerase [9]. Also, the expression of both PolB2 and Pol II are strongly induced upon DNA damage. However, whereas PolB2 appears to be responsible for most damage-induced mutations, the physiological role of Pol II in TLS is much less obvious [1].

The predicted structure of putative PolB2 mutasome has several intriguing features. One of the unexpected structural features is the mode of PolB2 and iRadA interaction, which mimics RadA-RadA (or Rad51-Rad51) interaction in the corresponding protein filament. A direct interaction between a DNA polymerase and a Rad51-family protein has not been previously identified. Nonetheless, the interaction via Rad51 oligomerization-like motif might be a common way for many proteins to bind Rad51/RadA as exemplified by Rad51-BRCA2 interaction [52], [70]. Another intriguing feature is the presence of a “shuttling” DNA sliding clamp binding motif in PolB2 mutasomes. In general, it is quite common for DNA polymerases to have clamp binding motifs. For example, four *E. coli* DNA polymerases, Pol II (B-family), Pol III α-subunit (C-family), Pol IV and Pol V (both Y-family) have a β-clamp binding motif [36], [54]. Most archaeal and eukaryotic DNA polymerases feature PCNA binding motifs. However, in this case the surprising observation was that not only some PolB2, but also iRadA sequences have the clamp binding motif in their C-terminal region. To our knowledge, this is the first time that a clamp binding motif has been detected in a recombinase homolog. Moreover, we found that the clamp binding motifs in iRadA are much more abundant than in PolB2, and only infrequently are present in both proteins from the same organism. This observation suggests that at least a fraction of PolB2 mutasomes function with DNA sliding clamp and that the clamp binding motif may “shuttle” between PolB2 and iRadA. Interestingly, bacterial PolB2 mutasomes have exclusively β-clamp binding motifs, indicating that upon transfer from archaea the PolB2 mutasomes had adjusted their clamp binding motifs to match the bacterial clamps.

In the case of Pol V, we have previously identified a RecA-NT motif in UmuC and showed that the UmuC-RecA interaction mirroring that in the RecA filament is supported by experiments [56]. Here, we constructed a full multimeric Pol V model (UmuD′_2_C-RecA) and contrasted it with the available experimental data related to protein-protein interactions within the Pol V mutasome. In addition to further corroborating the UmuC-RecA interaction mode, we identified several lines of evidence supporting the binding of the UmuC C-terminal region to UmuD′ and the asymmetric positioning of N-terminal arms of the UmuD′ dimer. A limitation of the Pol V model is that it represents a single static conformation, while it is known that Pol V is a highly dynamic system, and that ATP binding and hydrolysis plays a key role in conformational changes of the mutasome [66]. Unfortunately, although the novel DNA-dependent ATPase activity of Pol V has long been identified [71], the ATPase active site has not yet been mapped. To move beyond the static structure of Pol V and to explore its conformational dynamics using computational methods, experimental assignment of residues forming this novel active site is clearly needed.

**Fig. 8:**
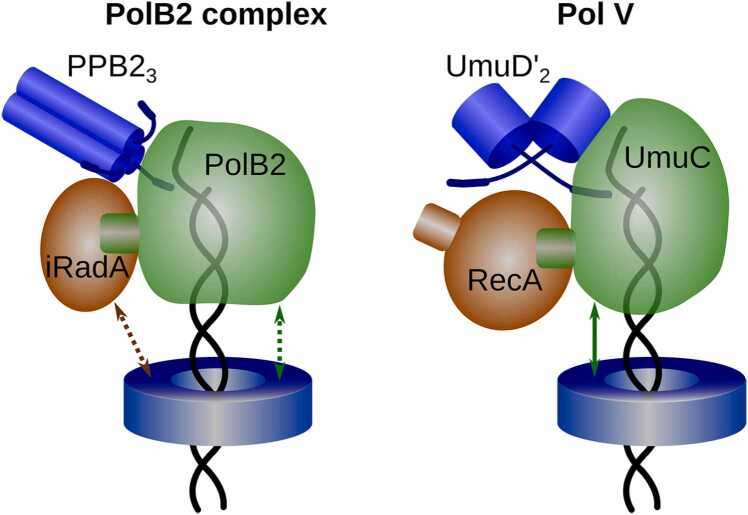
Schematic comparison of PolB2 and Pol V mutasome models. In both models, a catalytic polymerase subunit (PolB2 or UmuC) binds to a recombinase homolog (iRadA or RecA) through the motif that is analogous to the oligomerization motif within the corresponding filament (RadA/Rad51 or RecA). One of the subunits from the PPB2 trimer and the UmuD′ dimer inserts the N-terminal arm close to the respective polymerase active site and the bound DNA. A fraction of PolB2 complexes is predicted to bind DNA sliding clamp either through iRadA or PolB2 C-terminal motifs. Pol V mutasomes bind DNA sliding clamps through the motif in UmuC.

PolB2 and Pol V mutasomes, explored here, have obvious differences, but they also have striking parallels (Fig. 8). The two types of mutasomes employ catalytic polymerase subunits coming from different families (B and Y) that have inherently different fidelity. They also have different small subunits (PPB2 and UmuD′) with different stoichiometry within the mutasome. The PolB2 and Pol V mutasomes include homologous RecA/Rad51 family proteins, but they bind to different regions of corresponding catalytic subunits. Furthermore, many iRadA proteins feature clamp binding motif, whereas RecA does not have such a motif. Despite these differences, at the level of architectures, PolB2 and Pol V mutasomes share a number of common solutions. Both bind to a RecA/Rad51 family representative through the corresponding oligomerization motif. Pol V is known to act in concert with the sliding clamp [67], whereas here we show that some of the PolB2 complexes feature clamp binding motifs and should be able to bind a cognate DNA sliding clamp. Both types of mutasomes have an N-terminal arm of one subunit (UmuD′ in the case of Pol V and PPB2 in the case of PolB2) inserted deep into the catalytic subunit and close to its active site. Both the general parallels and the detailed structural features of the two mutasomes uncovered using AlphaFold models should facilitate comprehensive understanding of molecular mechanism of these intriguing molecular machines.

## Funding

Research Council of Lithuania (LMTLT) grant No. S-MIP-24–82.

## CRediT authorship contribution statement

**Česlovas Venclovas:** Writing – review & editing, Writing – original draft, Supervision, Project administration, Investigation, Funding acquisition, Conceptualization. **Kęstutis Timinskas:** Writing – review & editing, Visualization, Methodology, Investigation. **Albertas Timinskas:** Writing – review & editing, Visualization, Methodology, Investigation.

## Declaration of Competing Interest

The authors declare that they have no known competing financial interests or personal relationships that could have appeared to influence the work reported in this paper.

## Data Availability

Detailed data including annotation of PolB2 operons in both archaea and bacteria, structural models of PolB2 and Pol V complexes/subcomplexes and their annotation are available at https://doi.org/10.5281/zenodo.13087908.

## Footnotes

Appendix A Supplementary data associated with this article can be found in the online version at doi:10.1016/j.csbj.2025.01.010.
