# Supplementary material for: Common themes in architecture and interactions of prokaryotic PolB2 and Pol V mutasomes inferred from in silico studies
Source: Comput Struct Biotechnol J. 2025 Jan 16;27:401–10. doi: 10.1016/j.csbj.2025.01.010 (PMC11791011; doi:10.1016/j.csbj.2025.01.010)
Supplement: Supplementary file 3 — Supplementary material [file mmc3.pdf]

Common themes in architecture and interactions of prokaryotic PolB2 and Pol V mutasomes inferred from *in silico* studies

SUPPLEMENTARY FIGURES AND TABLES

Kęstutis Timinskas, Albertas Timinskas and Česlovas Venclovas

Institute of Biotechnology, Life Sciences Center, Vilnius University, Saulėtekio av. 7, Vilnius LT-10257, Lithuania

Corresponding author:

Česlovas Venclovas
Institute of Biotechnology, Life Sciences Center
Vilnius University
Saulėtekio 7,
Vilnius LT-10257, Lithuania

Phone: +370-5-223-4368
E-mail: ceslovas.venclovas@bti.vu.lt

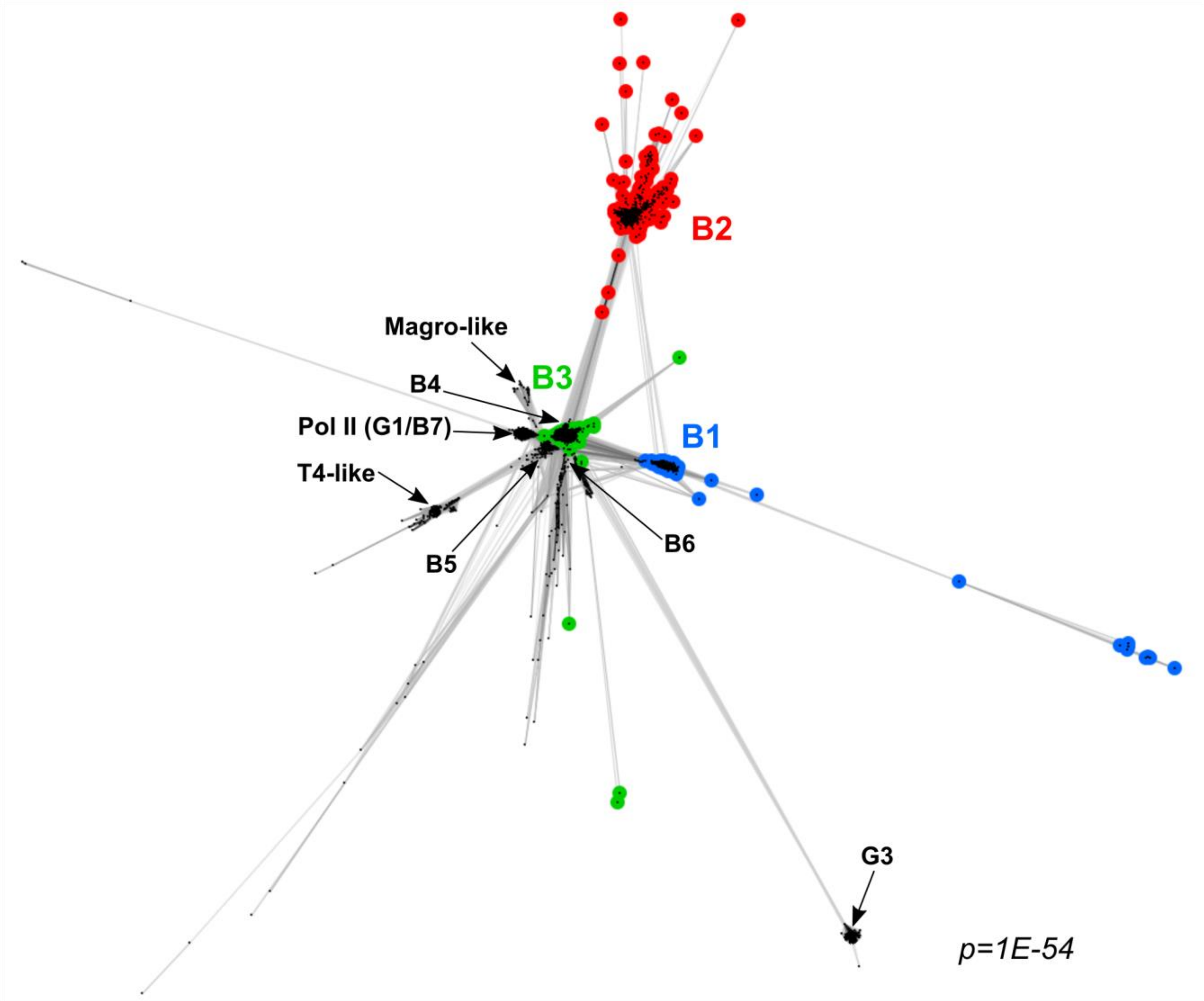

Supplementary Figure S1. Clustering of PolB2 (B2) polymerase homologs (filtered to 70% sequence identity) using CLANS. Lines connect similar proteins (p-value cutoff 1E-54). Only connected homologs are displayed. B-family polymerase clusters were assigned according to classification provided in Supplementary Table S5 of Kazlauskas D. *et al.* (reference 24 in the main text).

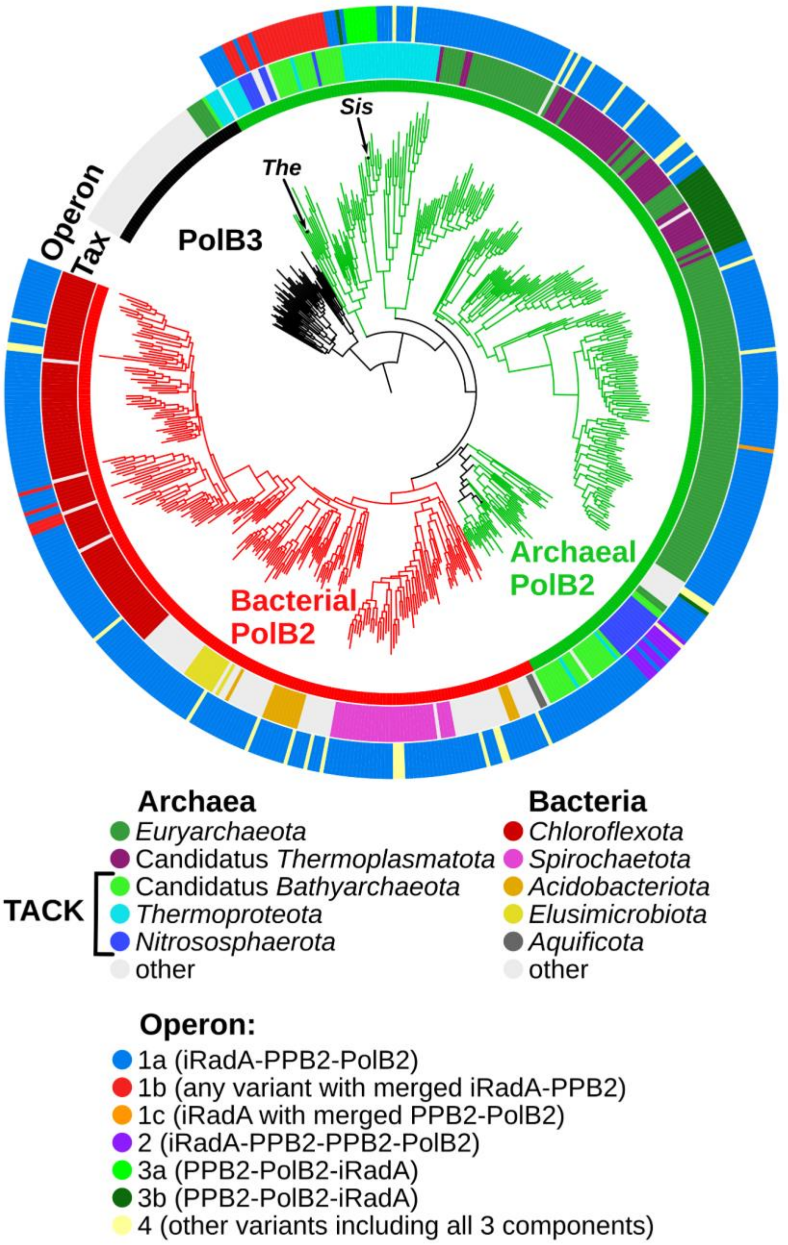

Supplementary Figure S2. Phylogenetic tree of PolB2 and 50 closest PolB3 homologs (same as in Figure 1). Tree branch colors: archaeal PolB2 (green), bacterial PolB2 (red), PolB3 outgroup (black). Middle ring ('Tax') shows the taxonomy (color legend provided below the tree). The outer ring is colored according to the operon group assigned to the respective PolB2.

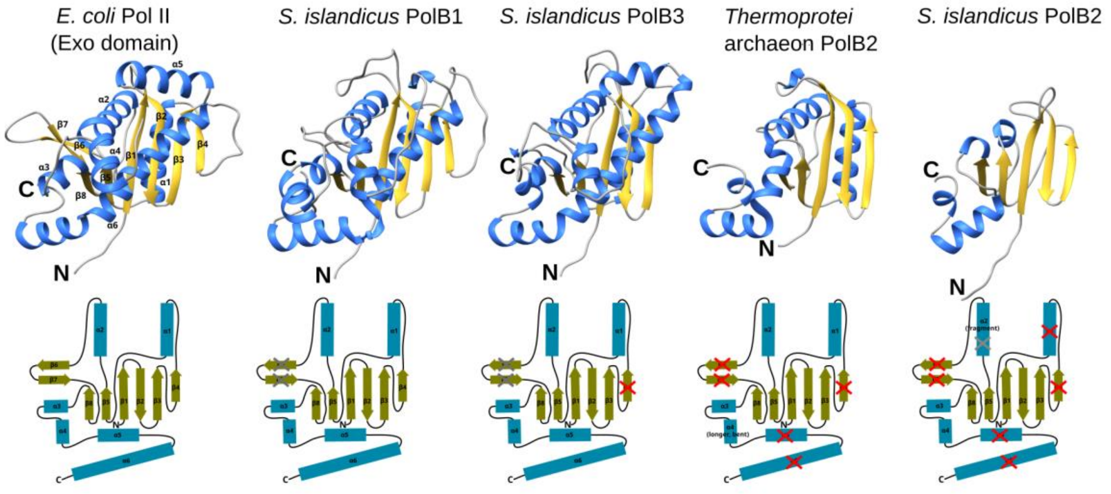

Supplementary Figure S3. Comparison of B family exonuclease domains.
E. coli Pol II exonuclease domain was cut from the experimental structure (PDB id: 3k59), all other domains were taken from monomer models in the EBI AlphaFold database: *S. islandicus* PolB1 (AFDB id: F0NBI0), *S. islandicus* PolB3 (AFDB id: F0NIP4), *Thermoprotei* archaeon PolB2 (AFDB id: A0A662H956), *S. islandicus* PolB2 (AFDB id: F0NED6). Gradual structural decay of the domain can be observed from left to right.
The three motifs containing active site residues are respectively located in β1, α2 and α6 secondary structure elements. β6-β7 motif is shorter in PolB1 and PolB3. Only Pol II and PolB1 have intact catalytic exonuclease sites (*E. coli* Pol II: D156, E158, D229, Y331, D335). *S. islandicus* PolB3 has 3 of the 5 residues mutated (not typical for PolB3). None of PolB2 has an intact active site.

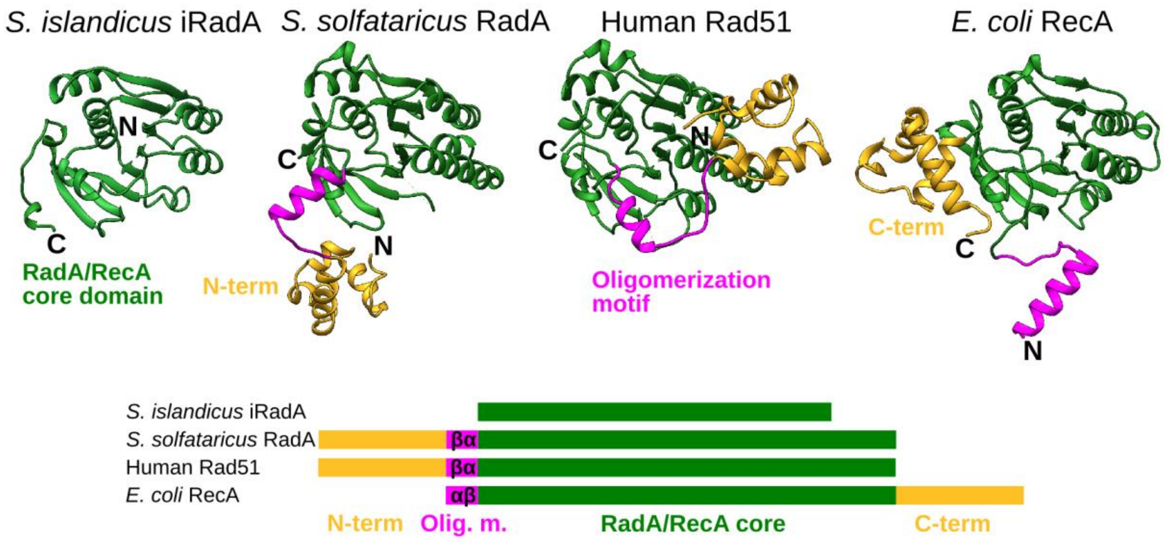

Supplementary Figure S4. Comparison of *S. islandicus* iRadA (AFDB model id: F0NED7), *S. solfataricus* RadA (PDB id: 2zub), human Rad51 (PDB id: 7c9a) and *E. coli* RecA (PDB id: 4twz). Corresponding structural components have the same color. Oligomerization motif is inverted in RadA (secondary structure element order: (β-α) compared to RecA (α-β)).

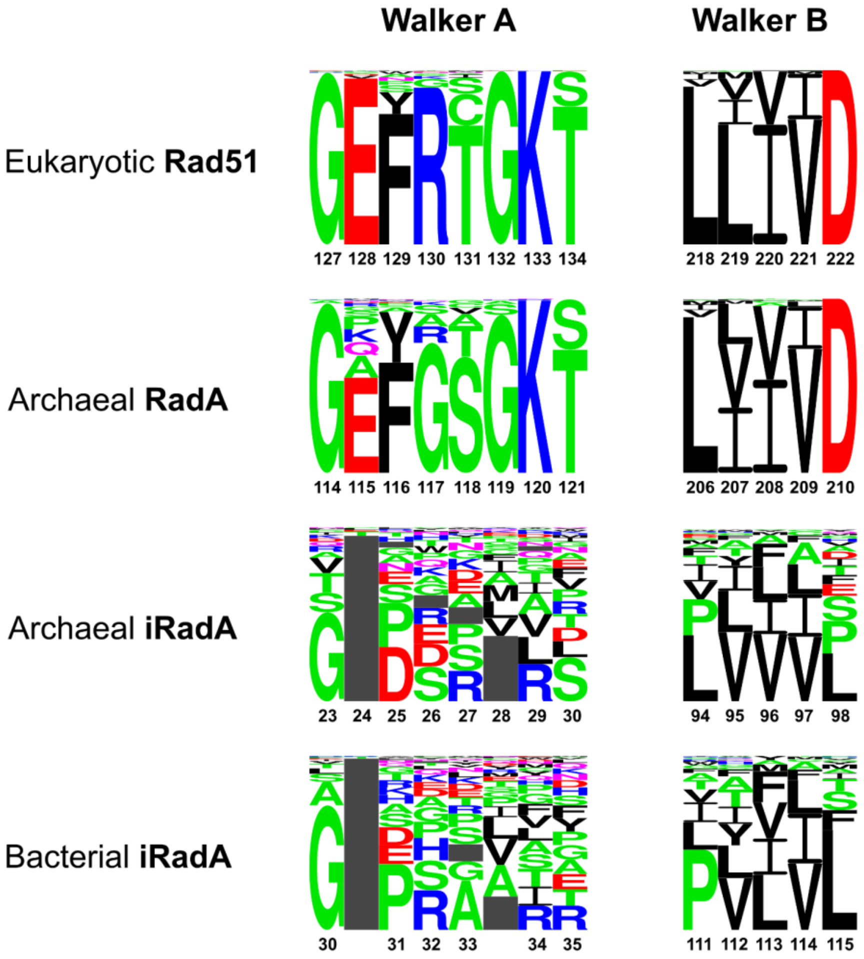

Supplementary Figure S5. Comparison of canonical ATP binding pocket motifs between eukaryotic Rad51, archaeal RadA, archaeal iRadA and bacterial iRadA. Positional numbering is based on corresponding representative sequences: human Rad51 (NCBI id: BAA03189), *S. solfataricus* RadA (NCBI id: AAK40589), *S. islandicus* iRadA (NCBI id: WP_012710852), *L. biflexa* iRadA (NCBI id: WP_012389130). Gray rectangles represent gaps in sequence alignment. Corresponding motifs have been determined based on structural alignment of representative structures: human Rad51 (PDB id: 7c9a), *S. solfataricus* RadA (PDB id: 2zub), *S. islandicus* iRadA (AFDB id: F0NED7), *L. biflexa* iRadA (AFDB id: B0ST20).

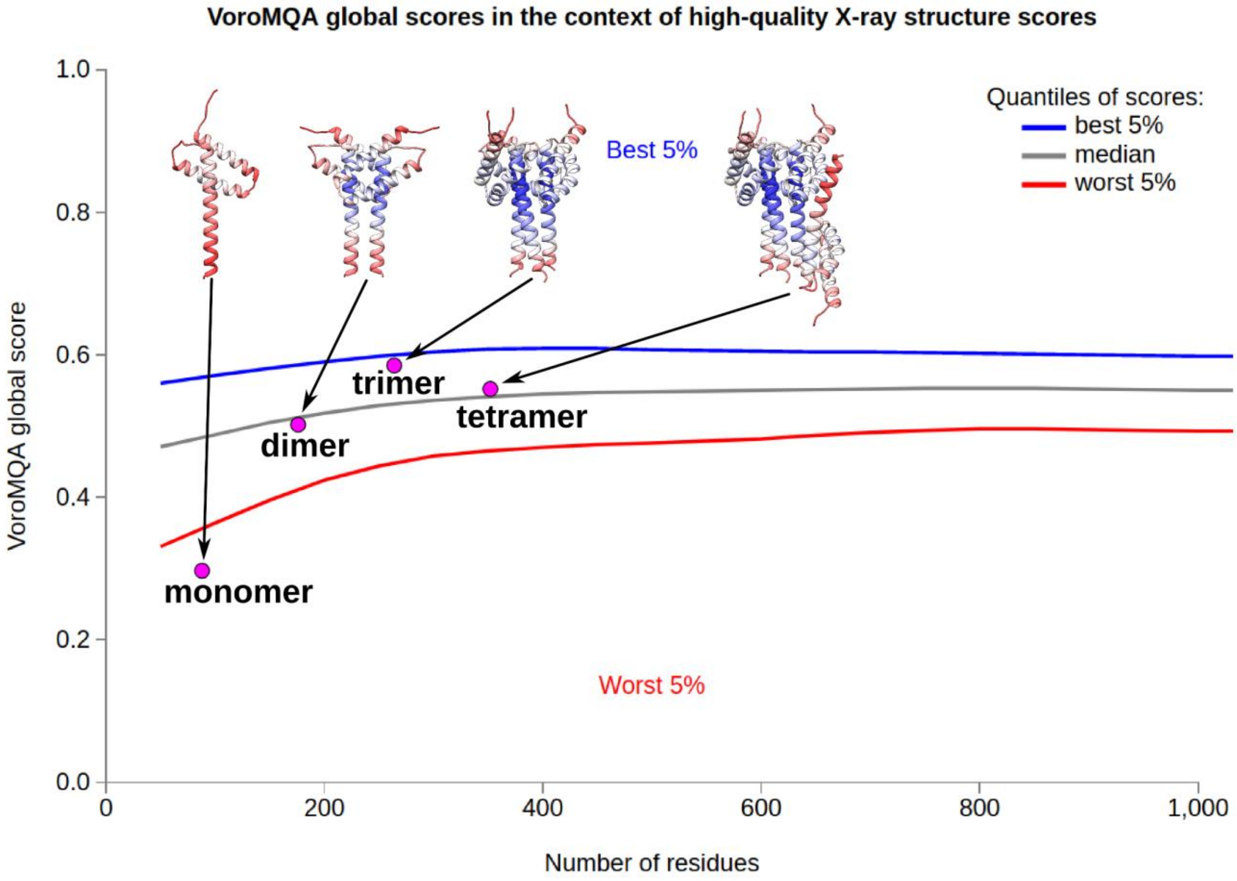

Supplementary Figure S6. VoroMQA score plot for *S. islandicus* PPB2 monomer, dimer, trimer and tetramer models (colored by averaged residue scores). AlphaFold-Multimer ranking scores: dimer - 0.48, trimer - 0,88, tetramer – 0.49; pTM scores: monomer – 0.79, dimer – 0.59, trimer – 0.89, tetramer – 0.58.

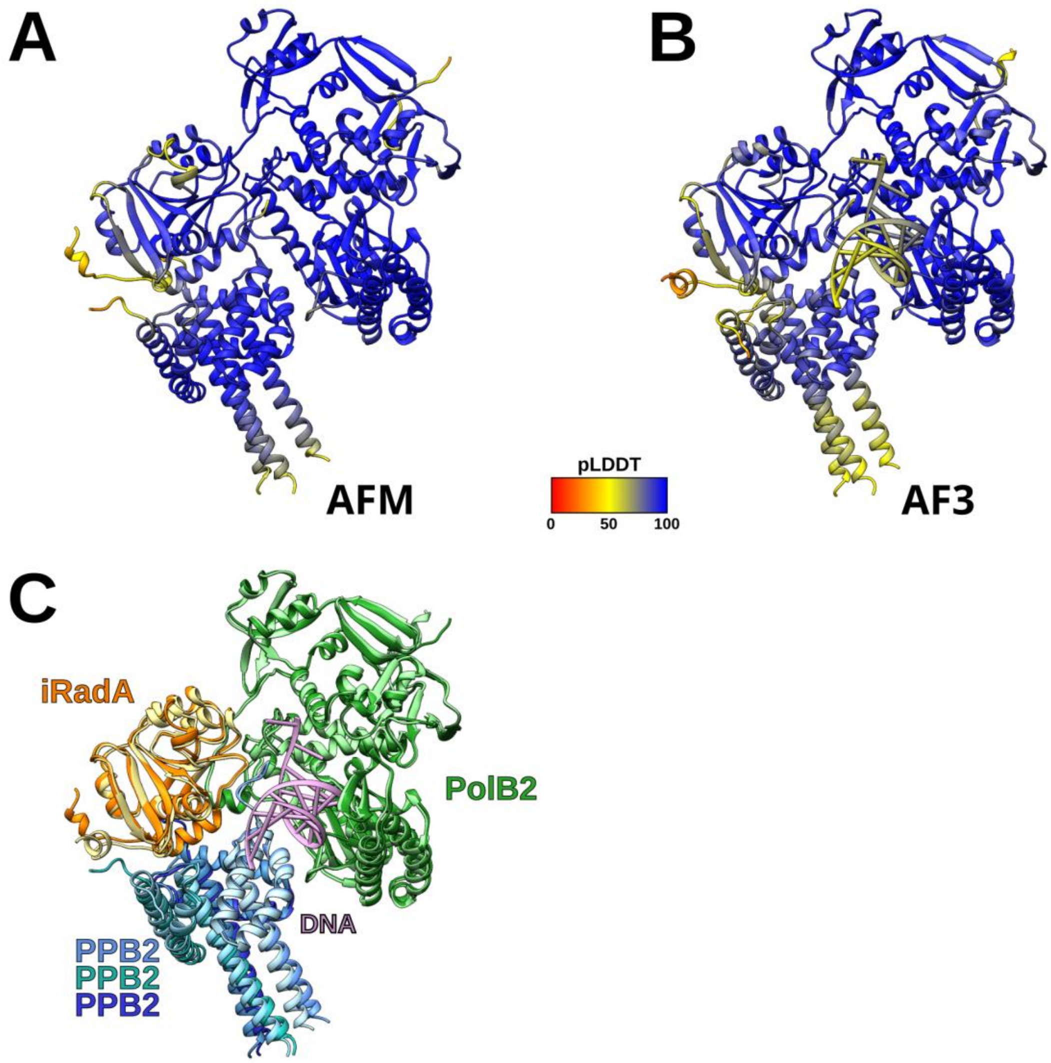

Supplementary Figure S7. Comparison of *S. islandicus* heteropentameric PolB2 complex models made with AlphaFold-Multimer (AFM) and AlphaFold3 (AF3). (A) AFM model colored by pLDDT; (B) AF3 model colored by pLDDT; (C) AFM model colored by protein components (same colors and orientation as in Figure 4A) and superimposed AF3 model colored similarly but in lighter colors. AFM model was made without DNA, AF3 model - with DNA (shorter variant of DNA present in *T. kodakarensis* PolB1 crystal structure, PDB id: 4k8z). AFM model confidence scores: pLDDT = 86, pTM = 0.88, ipTM = 0.85, ranking score = 0.85. AF3 model confidence scores: pLDDT = 85, pTM = 0.88, ipTM = 0.84, ranking score = 0.87.

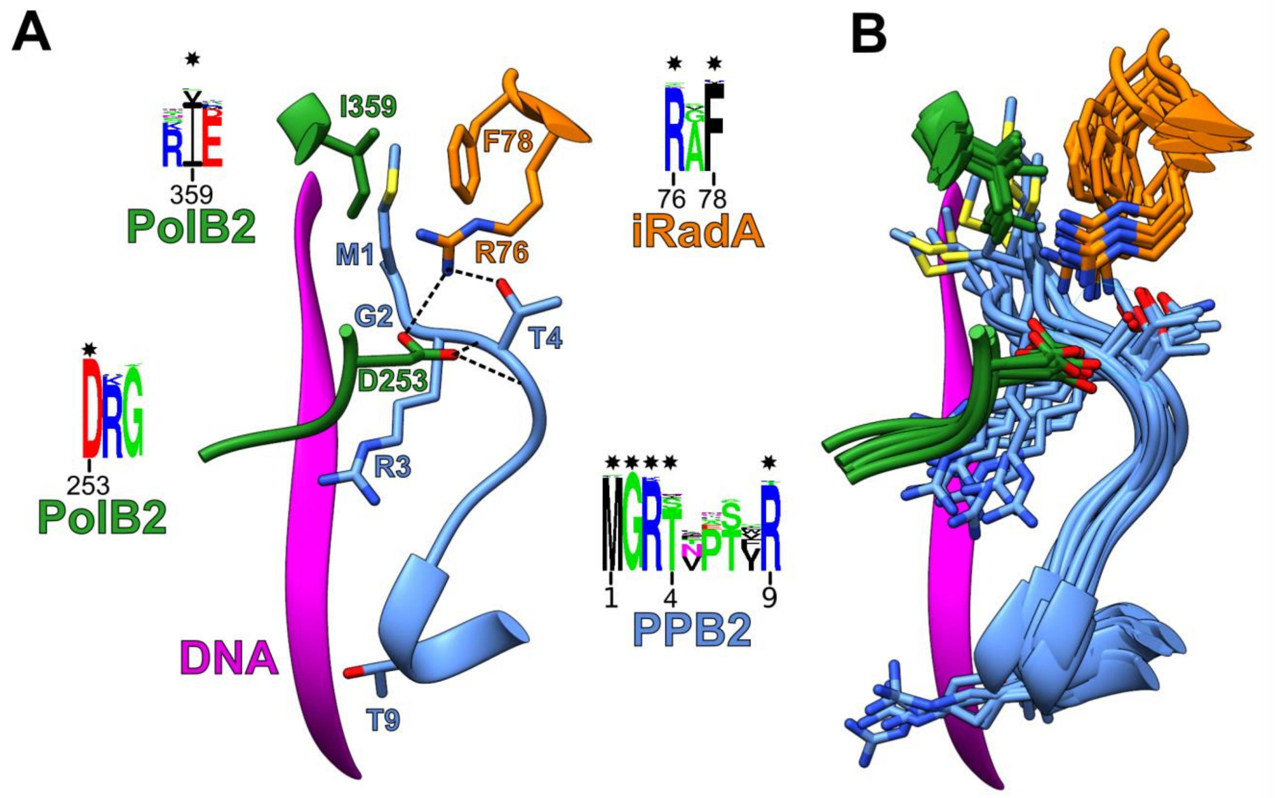

Supplementary Figure S8. Comparison of conserved interactions of PPB2 N-terminal tail in *S. islandicus* PolB2 and 10 additional PolB2 complex models from different organisms (5 archaeal, 5 bacterial): (A) *S. islandicus* model and corresponding information (same as in Figure 4B); (B) all 11 models superimposed.

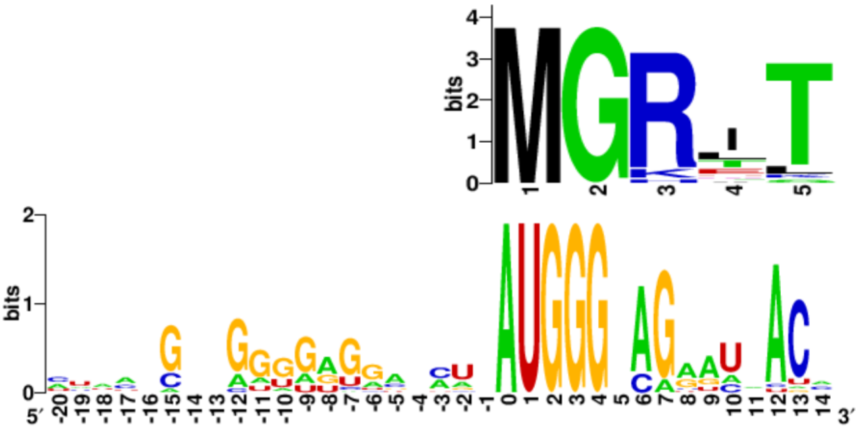

**KAA3644142.1**	AUAUCCucaAGGaGACUCCCAUGGGUCGCAACAGC
MBL7079594.1	UGUuuGAGGUGAACGAAGAAAUGGGAAGAGAUACA
MBO3803123.1	CGGAAGGAGGUGAGAGCCUUAUGGGAAGGAUAACC
MBT3285294.1	AUUAcGuuGuUGAUUUCAAGAUGGGAAGAGAAACA
MBT5642334.1	AUUAcGuuGuUGAUUUCAAGAUGGGAAGAGAAACA
MCB2171593.1	ACUAuGuuGuUGAUUUCAAGAUGGGAAGAGAGACA
MCJ7634486.1	ACGUuGaAGGUGAGAAGGCUAUGGGUAGAAUAACU
MCL4343870.1	UAACUguGAGGUGgGAAAGCAUGGGUAGAUUGACA
MCP8308408.1	CUAAAGGAGGgauGGAUAUAAUGGGGAGGAUUACC
MCP8311102.1	CUAAAGGAGGgauGGAUCUGAUGGGUAGAAUAACC
MCP8311737.1	CUAAAGGAGGgauGGAUCUGAUGGGUAGAAUAACU
MCP8322771.1	CUAAAGGAGGgauGGACCUGAUGGGUAGAAUAACU
MCW4037314.1	CUAAAGGAGGUGuGACUCUCAUGGGCCGUAUAACG
**MDC4225006.1**	CACCGGACCgGGAGGcGcUAAUGGGCCGCACCCUC
MDG6926953.1	UAACUAuGAGGUGgGAAAGCAUGGGUAGAUUGACA
MDG6928742.1	CGGCuGcAGGUGACCCGCUGAUGGGCAGGAUCACG
MDG6930495.1	UAACUAuGAGGUGgGAAAGCAUGGGUAGAUUGACA
MDG7012087.1	UCCAgGGAGGcGAGCCGCUGAUGGGGCGGGUGACC
MDH7555578.1	AUUGCCAuGGAGGUGAUCUUAUGGGGAGGAUAACC
**NAY82200.1**	UUACGGUuGGguGgGAUAAAAUGGGUAGGCUUACA
**NIV15258.1**	AAACACUCGAGGAGGgGuUUAUGGGAAAGACAUUA
**NMC12048.1**	AUCCACCGAAAGGAGaaaACAUGGGACAUACAAAA
NUM44842.1	CCCUUCCCCcGGAGacuuUCAUGGGCAAAACCACC
NWG08809.1	CUAAAGGAGGgGAGGACCUAAUGGGGAGAAUUACC
RLG87905.1	GCUAUGgGGAGGUGAGAAUCAUGGGCAGAUCCACG
RWX73647.1	AUUGGCAuGGAGGUGAUCUUAUGGGGAGGAUAACC

Supplementary Figure S9. Multiple mRNA sequences encoding merged iRadA-PPB2 proteins are enriched with Shine-Dalgarno (SD) motifs upstream of the start of PPB2 protein (potential AUG start codon is colored green). mRNA sequence motifs potentially interacting with ribosome 16S anti-Shine-Dalgarno region (antiSD) are colored red. Essential part of SD common for all organisms (5'-GGAGG-3') is shown in bold. Deviations from the Shine-Dalgarno consensus (5'-AGGAGGUGA-3') are shown as lowercase letters. Additionally, the topmost logo depicts the conservation of N-terminal residues of PPB2 within the merged protein (visually aligned above corresponding RNA sequences). Sequence names correspond to NCBI id's for iRadA-PPB2 proteins (bacterial sequence names are highlighted in bold, archaeal – no highlight).

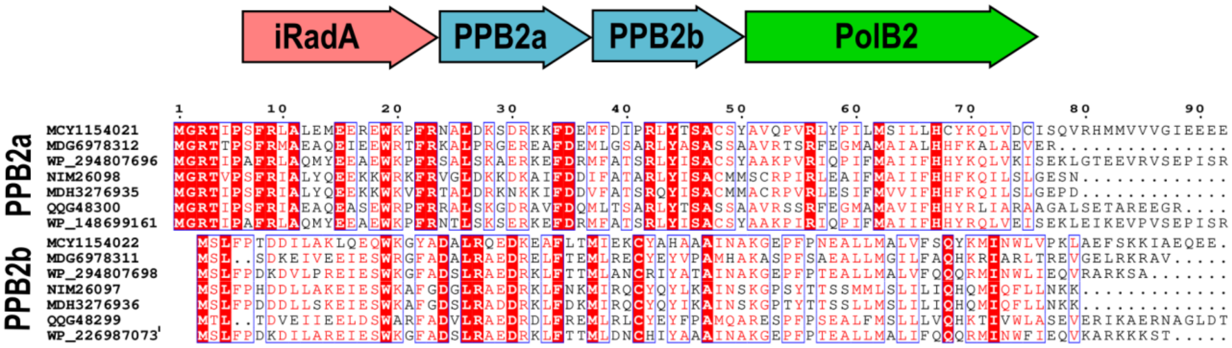

Supplementary Figure S10. Sequence difference between the first and the second PPB2 copies (PPB2a and PPB2b respectively) from the operons with duplicated PPB2. The displayed PPB2s have been selected to be taxonomically diverse. The PPB2a and PPB2b groups are displayed and colored separately. Alignment is colored by positional conservation using ESPript3 default coloring scheme.

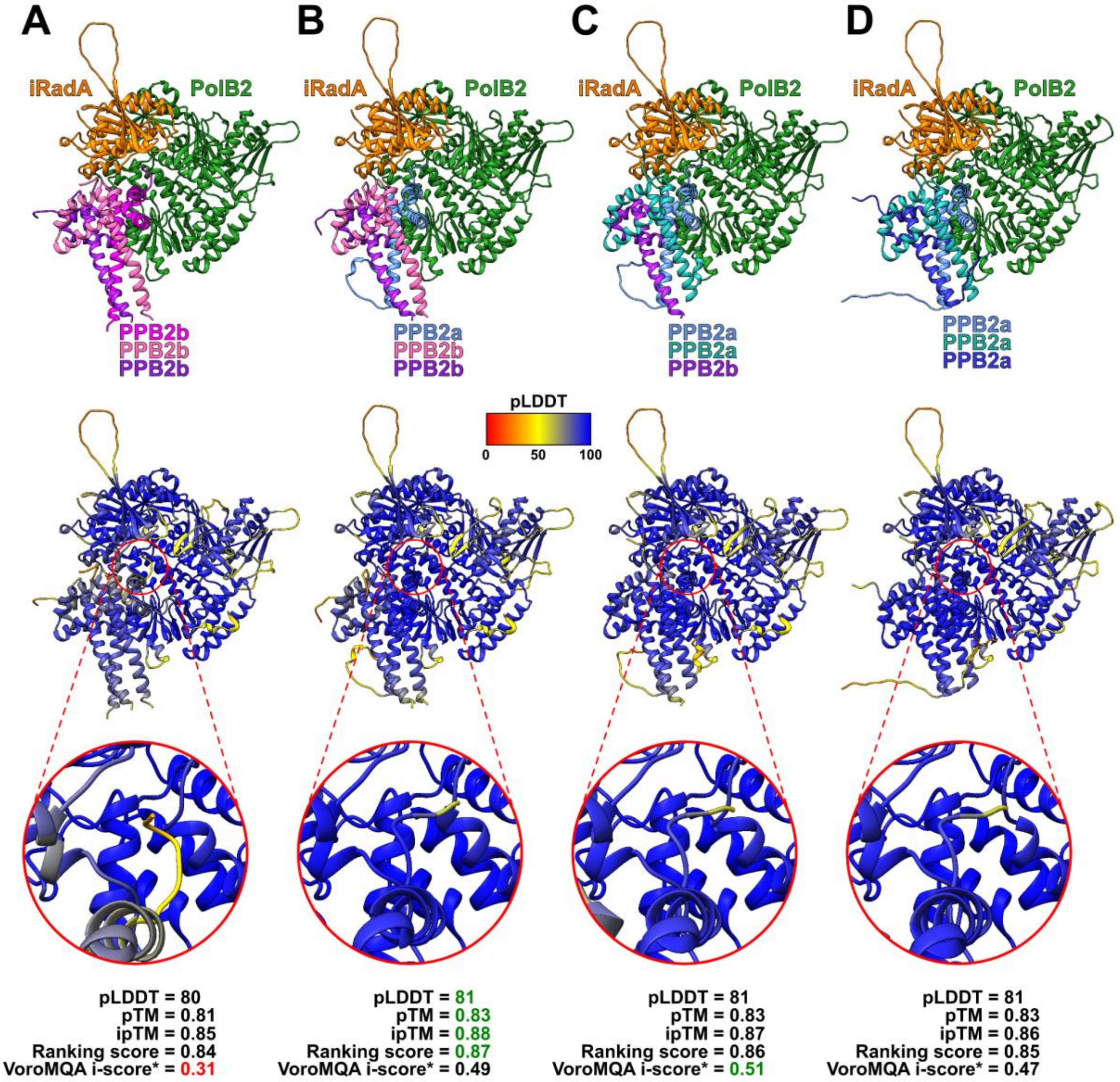

Supplementary Figure S11. Comparison of *Nitrosphaera sp.* heteropentameric PolB2 complex models with different combinations of PPB2a and PPB2b proteins. PPB2a has the conserved N-terminal ‘MGRT’ sequence motif, PPB2b does not have this motif (see Supplementary Figure S10). Sequence ids of the components: PolB – MCY1154023, iRadA – MCY1154020, PPB2a – MCY1154021, PPB2b – MCY1154022. (A) PolB2 + iRadA + 3 PPB2b; (B) PolB2 + iRadA + 2 PPB2b + PPB2a; (C) PolB2 + iRadA + PPB2b + 2 PPB2a; (D) PolB2 + iRadA + 3 PPB2a. For clarity, highly unstructured N-terminal tail of iRadA (residues 1-140) is not shown. For each complex, model structures are shown colored by components (top) and AlphaFold pLDDT scores (bottom). The zoomed in sections for each structure are focused on the PPB2 N-terminal arm that is inserted between PolB2 and iRadA. AlphaFold modeling scores are given for each corresponding model. VoroMQA i-score* corresponds to the interface score calculated between 8 N-terminal residues of PPB2 (the chain that inserts the N-terminus into the complex) and PolB2/iRadA (contacts to other PPB2 monomers were not included in the evaluation). Highest scores (before rounding) are highlighted in green, exceptionally low i-score* is highlighted in red.

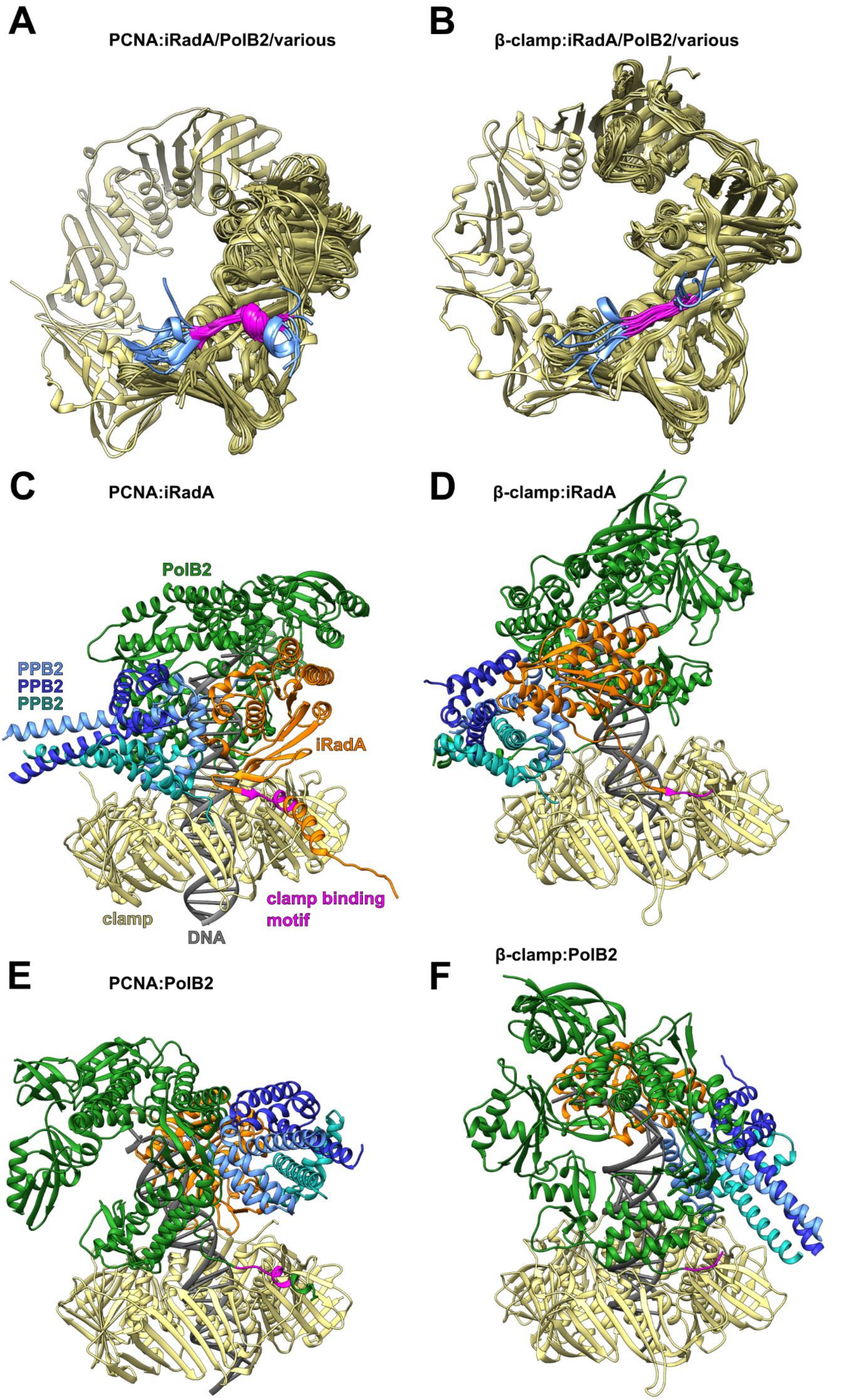
A
PCNA:iRadA/PolB2/various
B
β-clamp:iRadA/PolB2/various
C
PCNA:iRadA
PolB2
PPB2
PPB2
PPB2
iRadA
clamp binding
motif
clamp
DNA
D
β-clamp:iRadA
E
PCNA:PolB2
F
β-clamp:PolB2

Supplementary Figure S12. Comparison of clamp binding motifs with a bound clamp: (A) archaeal PCNA, (B) bacterial β-clamp. Clamp structures are colored dark khaki; identified clamp binding motifs are shown in magenta, other flanking residues from same proteins – blue; remaining parts of the structure are hidden. 'Various' – other proteins that interact with the clamp that have a known structure. All the displayed structures are the same as in Figure 6. Known structures and models included in the figures (unless indicated otherwise, id's denote NCBI sequence id's):
(A) PCNA:iRadA - *Natronobeatus ordinarius* WP_255192907:WP_255191964*, Halodesulfurarchaeum formicicum* WP_070365793:WP_071932650*, Halalkalicoccus paucihalophilus* WP_066383518:WP_066385307*, Halomarina salina* WP_247414337:WP_247417178*,* Candidatus *Lokiarchaeota* archaeon NHI91386:NHI94114;
PCNA:PolB2 - *Halanaeroarchaeum sulfurireducens* WP_050049189:WP_050048138*, Natronorubrum daqingense* WP_076582662:WP_076579486*, Natronorubrum sediminis* WP_090506199:WP_090505604*,* Candidatus *Bathyarchaeum* sp. MBN1785233:MBN1784956, Candidatus *Bathyarchaeota* archaeon MBL7168935:MBL7167843;
PCNA:various - *P. furiosus* PolB (PDB id: 3a2f), *S. solfataricus* Dpo4 (PDB id: 3fds), *P. abyssi* DP2 (PDB id: 6t7y), *A. fulgidus* RNase H2 (PDB id: 3p83), *P. furiosus* RFCL (PDB id: 1isq), *T. kodakarensis* FEN-1 (PDB id: 5dai), *A. fulgidus* FEN-1 (PDB id: 1rxz), *S. solfataricus* FEN-1 (PDB id: 2izo);
(B) β-clamp:iRadA - *Longilinea arvoryzae* WP_075072255:WP_075073596, *Pelolinea submarina* WP_116225279:WP_126440460*, Brevefilum fermentans* WP_087861171:WP_087862514*, Anaerolineae bacterium* NSW52789:NSW51109*, Anaerolineae* bacterium MDH3943149:MDH3944533;
β-clamp:PolB2 - *Anaerolineae* bacterium NSW52789:NSW51112, *Anaerolineae* bacterium TAK12634:TAK10710, *Leptonema illini* KAB2935358:KAB2932161*, Leptonema illini* WP_002774207:WP_002769645;
β-clamp:various - *E. coli* Pol II (PDB id: 3d1e), *E. coli* Pol III (PDB id: 3d1f), *E. coli* Pol IV (PDB id: 1ok7), *E. coli* UmuC (PDB id: 4k74), *M. tuberculosis* DnaE1 (PDB id: 8djq), *M. thermoresistibile* ImuB (PDB id: 8dj6), *M. tuberculosis* DinX (PDB id: 8dk9), *H. pylori* ligase (PDB id: 5frq).
(C-F) AlphaFold3 models of full PolB2-iRadA-PPB2x3-clamp-DNA complexes: (C) *Halalkalicoccus paucihalophilus* (NCBI id of PolB2: WP_066385324), representative model for PCNA:iRadA interaction; (D) *Brevefilum fermentans* (NCBI id of PolB2: WP_087862516), representative model for β-clamp:iRadA interaction; (E) Candidatus *Bathyarchaeota* archaeon (NCBI id of PolB2: MBL7167843), representative model for PCNA:PolB2 interaction; (F) *Anaerolineae* bacterium (NCBI id of PolB2: TAK10710), representative model for β-clamp:PolB2 interaction (for clarity, unstructured N-terminal 1-10 residues of PolB2 and 91-129 residues of each PPB2, modeled as an extra-long C-terminal α-helix bundle, are not shown). All four complexes are displayed in the same orientation of clamps and clamp binding motifs. Complexes are colored by components: PolB2 - green, iRadA - orange, PPB2 - different shades of blue, clamp - khaki, clamp binding motif - magenta, DNA - gray.

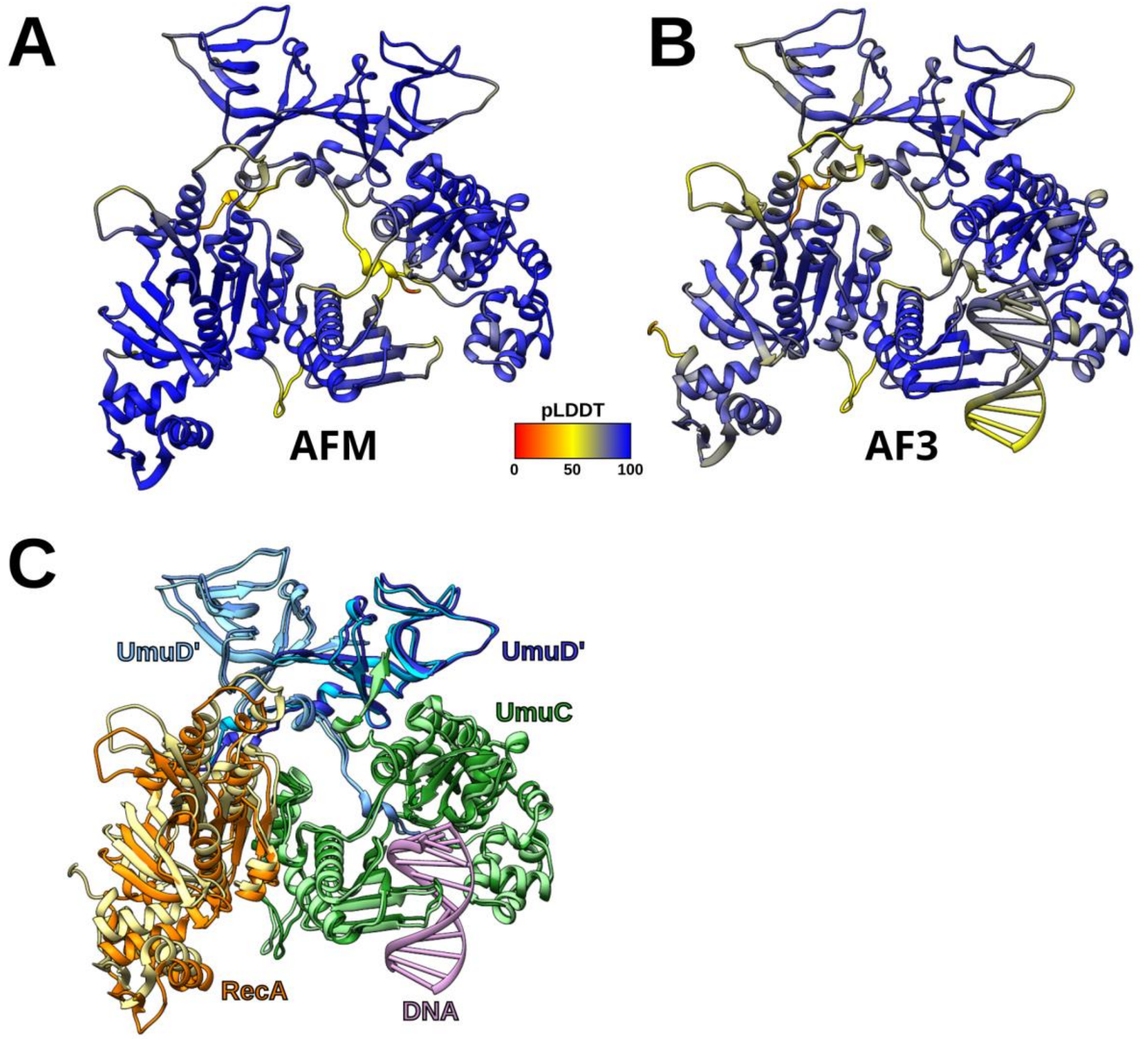

Supplementary Figure S13. Comparison of *E. coli* heterotetrameric Pol V complex models made with AlphaFold-Multimer (AFM) and AlphaFold3 (AF3). (A) AFM model colored by pLDDT; (B) AF3 model colored by pLDDT; (C) AFM model colored by protein components (same colors and orientation as in Figure 7A) and superimposed AF3 model colored similarly but in lighter colors. RecA N-terminal motif (1-37) and unstructured C-terminal residues (335-353) are not shown in either of the models. AFM model was made without DNA, AF3 model - with DNA (shorter variant of DNA present in *E. coli* Pol IV crystal structure, PDB id: 5yuu). AFM model confidence scores: pLDDT = 85, pTM = 0.62, ipTM = 0.64, ranking score = 0.63. AF3 model confidence scores: pLDDT = 80, pTM = 0.78, ipTM = 0.72, ranking score = 0.77.

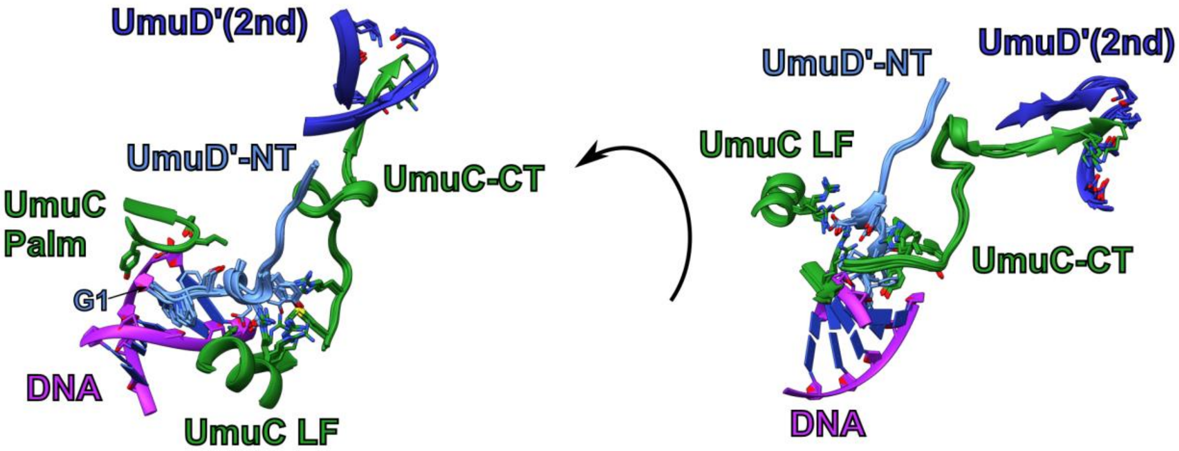

Supplementary Figure S14. Comparison of *E. coli* Pol V complex and 6 other homologous complex models. Only structural regions including the N-terminal part of UmuD' ('UmuD'-NT') and the C-terminal part of UmuC ('UmuC-CT') are displayed. The same structures are shown in two different orientations. 'LF' - 'Little finger' domain of UmuC polymerase. Part of superimposed DNA from *E. coli* Pol IV (PDB id: 5yuu) is displayed as purple ribbon.

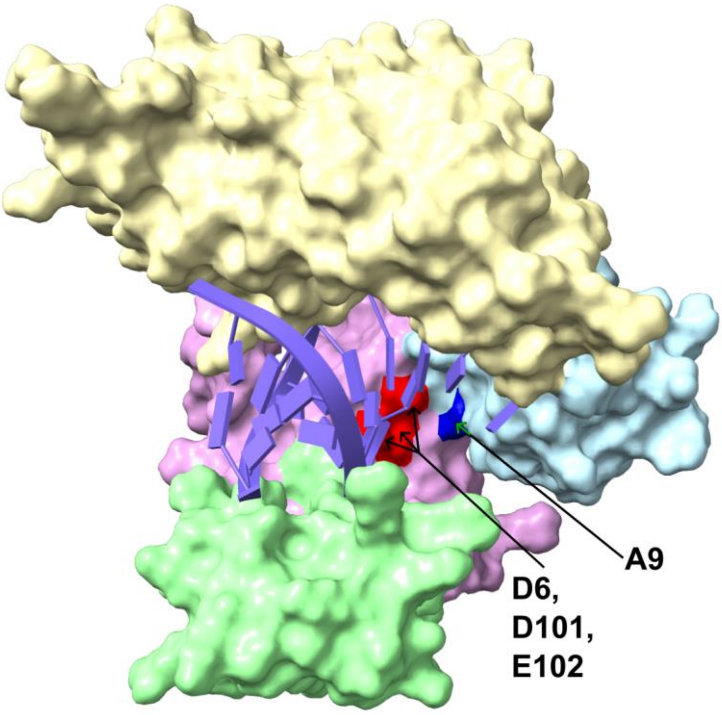

Supplementary Figure S15. Surface representation of *E. coli* UmuC structural model with the position of the Ala9 residue indicated. The UmuC A9 amino acid is colored in blue, polymerase catalytic site (D6, D101 and E102) is colored in red. Lighter surface colors denote UmuC structural domains: 'Palm' - plum, 'Fingers' – light blue, 'Thumb' – pale green, 'Little finger' – pale yellow. Part of superimposed DNA from *E. coli* Pol IV (PDB id: 5yuu) is displayed as a purple ribbon.

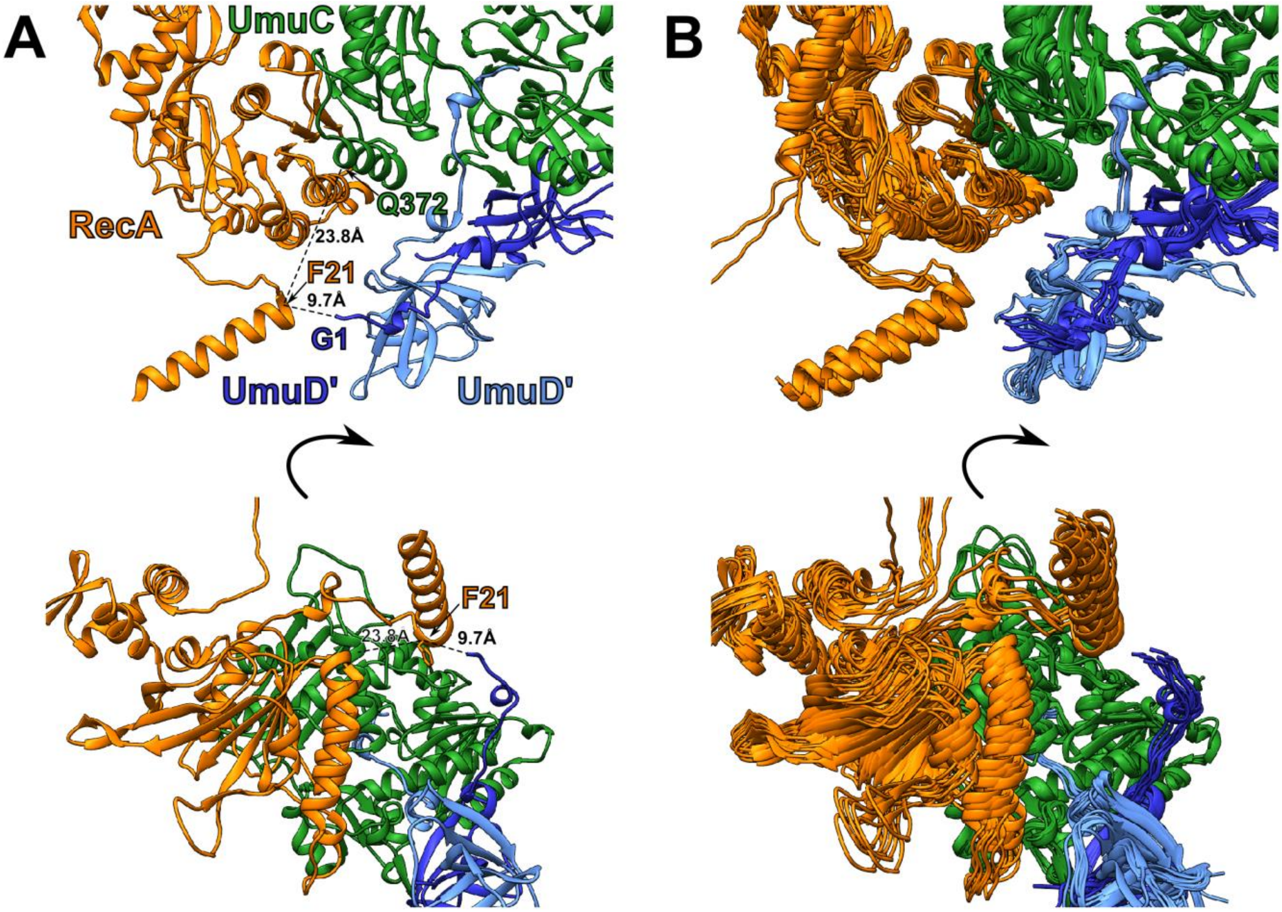

Supplementary Figure S16. Pol V models displayed in two different orientations. (A) *E. coli* Pol V model. Shortest distances between Cα atoms of RecA F21 and closest UmuC (Q372) and UmuD' (G1) residues are indicated with dashed lines. (B) Superposition of *E. coli* Pol V and six other homologous Pol V models.

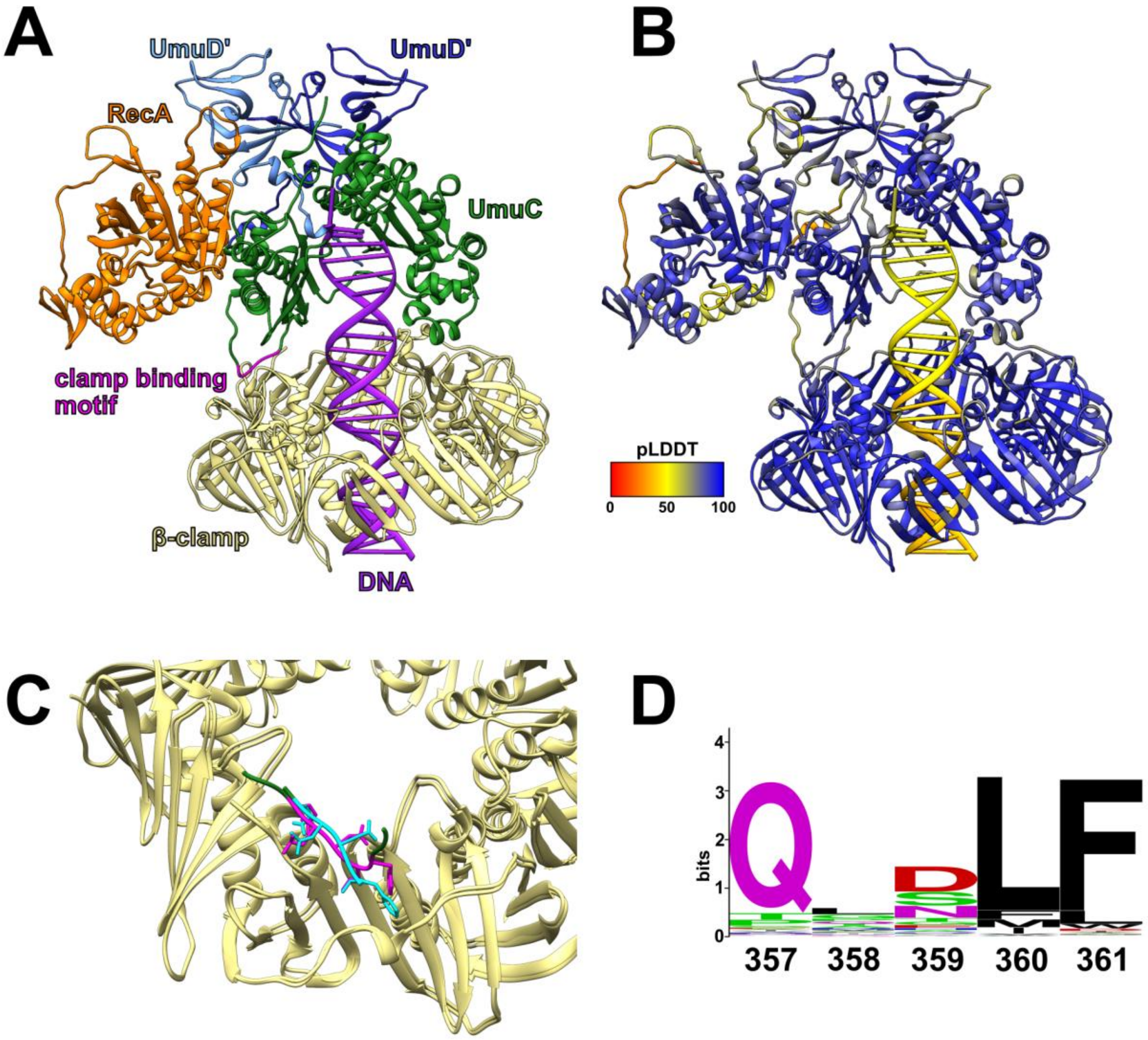

Supplementary Figure S17. AlphaFold3 model of a full *E. coli* Pol V complex with β-clamp and DNA: (A) model colored by components, UmuC - green, RecA - orange, UmuD' - shades of blue, β-clamp - khaki, clamp binding motif - magenta, DNA - purple; (B) model colored by AlphaFold pLDDT. (C) Comparison of modeled clamp binding motif (magenta) and the same motif (cyan) from *E. coli* crystal structure (β-clamp bound to clamp binding motif of UmuC, PDB id: 4k74). Structures were superimposed based on the β-clamps. (D) Clamp binding motif logo of UmuC homologs. Position numbering is based on *E. coli* UmuC sequence (motif: QLNLF).

Supplementary Table S1. Most abundant protein domains (PFAM v.37.0) encoded in the neighborhood of *polB2* within bacterial and archaeal genomes. Protein-coding genes in ±5 positions around *polB2* were compared to all PFAM hmm profiles using HMMER and only the best hits for each protein were retained. Listed are all hits (including PFAM domain ids) with E-value better than 1e-5. PolB2 (polymerase domain), PPB2 (PPB2 domain), iRadA (iRadA domain), orc1/cdc6, SRAP, DUF72 and LexA hmm profiles were constructed from just the proteins/domains identified in the neighborhood of *polB2*.

		**PolB2 operon group**							
#	**Protein domain family**	**Total**	**1a**	**1b**	**1c**	**2**	**3a**	**3b**	**4**
1	PolB2	1823	1375	57	1	58	50	95	187
2	PPB2	1885	1380	57	1	116	50	95	187
3	iRadA	1823	1375	57	1	58	50	95	187
4	orc1/cdc6 (PF09079+PF22606+PF13191/PF13401)	364	296	11	0	1	0	0	56
5	SRAP (PF02586)	134	121	0	0	0	0	3	10
6	ABC_tran PF00005.31	134	100	7	0	11	2	3	11
7	GerE PF00196.23	131	127	2	0	0	0	0	2
8	DUF72 (PF01904)	119	90	6	0	12	0	0	11
9	LexA (PF01726 +PF00717)	108	76	1	0	0	0	0	31
10	MarR_2 PF12802.11	79	50	1	0	0	0	0	28
11	Response_reg PF00072.28	69	67	0	0	0	0	0	2
12	Radical_SAM PF04055.25	65	46	2	0	0	9	0	8
13	Acetyltransf_1 PF00583.29	59	50	0	0	1	0	0	8
14	2-Hacid_dh_C PF02826.23	57	6	0	0	0	0	45	6
15	IMS PF00817.24	56	4	0	0	0	0	46	6
16	Lactamase_B PF00753.31	54	13	3	0	0	3	32	3
17	RNase_H_2 PF13482.10	50	12	2	0	0	0	0	36
18	MFS_1 PF07690.20	49	32	7	0	2	5	0	3
19	tRNA-synt_1 PF00133.26	48	2	0	0	0	0	42	4
20	HTH_20 PF12840.11	35	16	1	0	1	0	0	17

Supplementary Table S2. Comparison of average model confidence scores of PolB2+(x*PPB2a+y*PPB2b)+iRadA oligomer models (for operons with duplicated PPB2) with different combinations of PPB2a and PPB2b. Only models with three copies of PPB2 were included, x+y=3. Models of full complexes from five different organisms have been included in the analysis (any pair of selected PPB2a proteins has less than 60% sequence identity). Error values were calculated as 95% confidence score, based on t-distribution of sample standard deviation. VoroMQA i-score* corresponds to the interface score calculated between 8 N-terminal residues of PPB2 (the chain that inserts the N-terminus into the complex) and PolB2/iRadA (contacts to other PPB2 monomers were not included in the evaluation). This score highlights the difference between models that have at least one copy of PPB2a (with the typical N-terminal tail) and models that only have copies of PPB2b.

**PPB2 component**	**AF pLDDT**	**AF pTM**	**AF ranking score**	**VoroMQA score**	**VoroMQA i-score**	**VoroMQA i-score***
3xPPB2a	80±8	0.78±0.16	0.79±0.10	0.53±0.03	0.60±0.06	0.37±0.13
1xPPB2a+2xPPB2b	**83±7**	**0.80±0.16**	**0.83±0.09**	0.54±0.02	0.60±0.04	**0.39±0.13**
2xPPB2a+1xPPB2b	81±7	0.79±0.17	0.81±0.10	0.54±0.02	0.60±0.04	0.38±0.13
3xPPB2b	81±6	0.76±0.15	0.77±0.06	0.53±0.03	0.59±0.06	0.20±0.09
